# Characteristic Cytokine and Chemokine Profiles in Encephalitis of Infectious, Immune-Mediated, and Unknown Aetiology

**DOI:** 10.1371/journal.pone.0146288

**Published:** 2016-01-25

**Authors:** Benedict D. Michael, Michael J. Griffiths, Julia Granerod, David Brown, Nicholas W. S. Davies, Ray Borrow, Tom Solomon

**Affiliations:** 1 The Walton Centre NHS Foundation Trust, Liverpool, United Kingdom; 2 The Institute of Infection and Global Health, University of Liverpool, Liverpool, United Kingdom; 3 NIHR Health Protection Research Unit in Emerging and Zoonotic Infections, University of Liverpool, Liverpool, United Kingdom; 4 Alder Hey Children’s NHS Foundation Trust, Liverpool, United Kingdom; 5 Public Health England, London, United Kingdom; 6 Influenza and measles laboratory, IOC, Fiocruz, Rio de Janeiro, Brazil; 7 Chelsea and Westminster NHS Foundation Trust, London, United Kingdom; 8 Vaccine Evaluation Unit, Public Health England, Manchester, United Kingdom; University of Leuven, Rega Institute, BELGIUM

## Abstract

**Background:**

Encephalitis is parenchymal brain inflammation due to infectious or immune-mediated processes. However, in 15–60% the cause remains unknown. This study aimed to determine if the cytokine/chemokine-mediated host response can distinguish infectious from immune-mediated cases, and whether this may give a clue to aetiology in those of unknown cause.

**Methods:**

We measured 38 mediators in serum and cerebrospinal fluid (CSF) of patients from the Health Protection Agency Encephalitis Study. Of serum from 78 patients, 38 had infectious, 20 immune-mediated, and 20 unknown aetiology. Of CSF from 37 patients, 20 had infectious, nine immune-mediated and eight unknown aetiology.

**Results:**

Heat-map analysis of CSF mediator interactions was different for infectious and immune-mediated cases, and that of the unknown aetiology group was similar to the infectious pattern. Higher myeloperoxidase (MPO) concentrations were found in infectious than immune-mediated cases, in serum and CSF (p = 0.01 and p = 0.006). Serum MPO was also higher in unknown than immune-mediated cases (p = 0.03). Multivariate analysis selected serum MPO; classifying 31 (91%) as infectious (p = 0.008) and 17 (85%) as unknown (p = 0.009) as opposed to immune-mediated. CSF data also selected MPO classifying 11 (85%) as infectious as opposed to immune-mediated (p = 0.036). CSF neutrophils were detected in eight (62%) infective and one (14%) immune-mediated cases (p = 0.004); CSF MPO correlated with neutrophils (p<0.0001).

**Conclusions:**

Mediator profiles of infectious aetiology differed from immune-mediated encephalitis; and those of unknown cause were similar to infectious cases, raising the hypothesis of a possible undiagnosed infectious cause. Particularly, neutrophils and MPO merit further investigation.

## Introduction

Encephalitis is a neurological emergency, which presents as headache, cognitive and behavioural disturbance, sometimes with focal neurological signs; many cases progress to seizures, coma and death [1]. The most common aetiologies are infectious, typically viruses, or immune-mediated processes, which are often associated with specific antibodies [2,3]. Whilst there may be overlap in the clinical presentations of these different aetiologies, early distinction is critical, as specific antiviral or immunosuppressive treatment is required depending on the cause [1,4]. Moreover, delays in starting this specific treatment are associated with increased morbidity and mortality [5–8]. For example, in the most common sporadic viral encephalitis, that due to herpes simplex virus (HSV), 70% of patients die without treatment, and this is reduced to 10–30% with early aciclovir [6,9]. Despite the increasing recognition of immune-mediated causes of encephalitis, the aetiology of encephalitis remains unknown in 15–60% in most studies [10].

There is mounting evidence that the host inflammatory response, driven by cytokines, chemokines and associated mediators, may play a pivotal role in the pathogenesis of encephalitis [11–14], and that some may represent biomarkers of subtypes of disease within a given aetiology [15,16]. Indeed mediator profiles have been found to differ between aetiologies in other diseases of central nervous system (CNS) inflammation [17,18]. However, whether these profiles differ between infectious and immune-mediated encephalitis has not been established [19].

Earlier work has demonstrated the importance of considering the significance of any identified mediators in the pathogenesis of disease in the context of leucocyte subsets within the CNS [20]. The early influx of neutrophils and subsequent necrosis recognised in HSV encephalitis is not reported in immune-mediated cases, suggesting that there may be some significant differences, perhaps for mediators associated with neutrophil chemotaxis or degranulation, such as myeloperoxidase (MPO) [21,22]. An improved understanding of the pathophysiology of inflammation in encephalitis may have implications for optimising diagnostic tests and guiding the use of adjunctive immune-modulatory therapies, such as those used in other neurological inflammatory diseases [23,24].

Therefore, we conducted a study to examine the mediator profiles in the cerebrospinal fluid (CSF) and serum of patients with acute encephalitis to address two questions. Firstly, does the mediator profile differ between those of infectious and immune-mediated aetiologies; and secondly might characteristic mediator profiles suggest whether those of unknown aetiology are more likely to be infectious or immune-mediated.

## Materials and Methods

### Patients

We analysed samples available from adult patients in a previous prospective clinical and diagnostic study- the Health Protection Agency (now Public Health England) Aetiological Study of Encephalitis in England; this recruited 203 patients from 24 centres which each recruited over 2 years (2005–2008); methods have been described in detail [2]. In brief, the case definition included any person, of any age admitted to hospital with encephalopathy (altered consciousness that persisted for >24 hours, including lethargy, irritability, or a change in personality and behaviour) and with ≥2 of: fever or history of fever (≥38°C); seizures and/or focal neurological findings (with evidence of brain parenchyma involvement); CSF pleocytosis (>4cells/μL); and electroencephalographic findings or neuroimaging suggestive of encephalitis. All patients underwent an extensive panel of routine clinical and additional CSF testing for RNA, DNA and antibodies to establish the aetiology. The investigations were guided by immune status, travel history and, when no aetiology was established, further second-line testing as directed by an expert panel review (Tables 1 and 2). Patients were catagorised into aetiological groups as ‘Infectious’ if a causal pathogen was identified, as ‘Immune-mediated’ if a causal antibody was identified or they met the study definition for demyelinating illness, including acute disseminated encephalomyelitis (ADEM), and as ‘Unknown’ if they did not meet either of these groups. Serum and CSF samples from patients in the cohort were used for this study if, after all the diagnostic tests had been completed and an archived aliquot stored, >50υL was available for testing.

**Table 1:** First line testing for all patients with encephalitis recruited through the prospective HPA Study of the Aetiology of Encephalitis in England*.

Sub-group	Investigation
**If immunocompetent**	*Routine CSF PCR*:
	Herpes simplex virus 1/2
	Varicella zoster virus
	Enterovirus
	Parechovirus
	Adenovirus
	Human herpesvirus 6/7 (<30 years)
	Consider other tests depending on clinical features†
	*Routine serology*:
	Mumps or measles
	Influenza A or B
**If immunocompromised**	*CSF PCR As for immunocompetent*, *and consider*:
	Cytomegalovirus
	Epstein-Barr virus
	Human herpesvirus-6/7
	JC virus
	Lymphocytic choriomeningitis virus
	JC virus
	HIV Serology
**If travelled abroad**	*CSF PCR As for immunocompetent*, *and consider*:
	Arboviruses (Japanese encephalitis, dengue, tick-borne encephalitis, Murray Valley encephalitis, St Louis encephalitis)
	Nipah virus
	Poliomyelitis
	Rabies
	West Nile virus

*Algorithm assumes appropriate investigations were also done when clinically indicated to exclude bacterial, fungal, and parasitic infections.

^†^For cervical lymphadenopathy consider cytomegalovirus and Epstein-Barr virus; for respiratory illness consider influenza A and B; for parotitis and orchitis consider mumps.

**Table 2:** Additional pathogens investigated in patients in whom panel 1 testing was negative as determined by the expert review panel for the HPA Study of the Aetiology of Encephalitis in England.

Group	Pathogen
Viral	Cytomegalovirus, Epstein-Barr virus, flaviviruses, hepatitis viruses, human T-cell lymphotropic virus, lymphocytic choriomeningitis virus, parainfluenza virus, parvovirus B19, poliovirus, rabies virus, respiratory syncytial virus
Bacterial	*Bacillus anthracis*, *Bartonella henselae*, *Chlamydophila psittaci*, *Chlamydia trachomatis*, *Legionella pneumophila*, *Leptospira* spp, *Listeria monocytogenes*, *Borrelia burgdorferi*, *Mycoplasma pneumoniae*, *Mycobacterium tuberculosis*, *Salmonella* spp, *Streptococcus pneumoniae*, *Streptococcus pyogenes*
Rickettsial	*Coxiella burnetii*, *Rickettsia rickettsia*
Parasitic	*Toxoplasma gondii*
Fungal	*Histoplasma capsulatum*

Patients provided written informed consent, the procedure for which was approved in the HPA study by the North and East Devon Multicentre Research Ethics Committee (Reference: 05/Q2102/22). This sub-study was approved by the HPA Encephalitis Study Steering Group and also by the Pan-Manchester Research and Development Group for the University of Manchester

### Measurement of mediators

Serum and CSF samples were collected at recruitment and stored at -80°C. Thirty mediators (cytokines, chemokines and associated mediators) were assessed using cytometric bead array (Procarta Affymetrix Italy). A further panel for eight matrix metalloproteinases was performed using the same technique where additional volumes were available. Mediators were identified from previous literature and chosen to reflect, in part:

- Leucocyte proliferation and differentiation (granulocyte-colony stimulating factor [G-CSF], granulocyte-macrophage-colony stimulating factor [GM-CSF], granzyme B, myeloperoxidase, leptin and eSelectin);
- Leucocyte chemotaxis (CCL2 [monocyte chemotactant protein 1], CCL3 [monocyte inflammatory protein 1α], CCL5 [regulated on activation normal T cell expressed and secreted], CXCL9, CXCL10 [inducible protein 10]);
- Adhesion molecules (vascular cell adhesion molecule [VCAM], intracellular adhesion molecule [ICAM]);
- Markers of BBB permeability (vascular endothelial growth factor [VEGF]α, and matrix metalloproteinases [MMP] 1,2,3,7,8,9,12, and 13);
- Antiviral and related peptides (interferon [IFN] α,β,ο,γ tumour necrosis factor [TNF]α and its soluble receptors [TNFR1 and TNFR2] and TNFα-related apoptosis inducing ligand [TRAIL]);
- Broadly pro-inflammatory interleukins (IL-1α, 1β, 6, 8, and 17α); and
- Broadly anti-inflammatory interleukins (IL1-receptor antagonist [IL1RA], IL-4, and IL-10).

Fluorescence intensity was determined using a Bio-Rad platform (BioPlex Manager 4.1, Bio-Rad, UK). Standards and samples were analysed in duplicate and mean value used. Standard curves were adjusted at the points of fluorescence intensity saturation, generating a sigmoid curve with 6–8 fluorescence points, as described [17]. To avoid undetectable levels or missing data bias, only mediators detected in >80% were included in analyses, as described [11].

### Statistics

Freeze/thaw cycles were minimised, typically to 2–3, in the central laboratory. Nevertheless, to minimise any potential impact of any possible variation in sample storage, concentrations were median-centred and normalised for each patient, using established methodology [11,17]. Therefore, the concentration of each mediator was expressed and analysed as a value relative to the median concentration of all of the mediators in that patient sample [11,17]. These median-centered concentrations of each mediator were compared for statistically significant differences between the aetiological groups rather than comparing against normative data, as it was the power of mediators to distinguish between the causes of encephalitis that was the focus of this paper. Overall mediator data for all aetiologies underwent a one-way hierarchical cluster analysis. Then mediator data from each aetiological group underwent nearest neighbour analysis, using Pearson’s correlation coefficient, to generate proximity matrices (representing the strength of correlation between mediators)(SPSS 2011); from these, heat-maps were generated (SigmaPlot, Systat Software,USA), as described [17,25]. This generates a visual representation of the interaction of all mediators with each other in each aetiological group.

The Mann-Whitney U test was used for categorical variables and Kendall’s rank for continuous variables (SPSS 2011). Step-wise discriminant function analysis was used to develop a multivariate model, which underwent a leave-one-out cross-validation to protect against type 1 error; the power of each mediator is expressed using Wilk’s Lambda coefficient, from perfect discriminatory power (0.0) to none (1.0). This assisted by generating a model based not just on absolute concentrations of selected mediator(s), but also with a numerical weighting applied to selected mediator(s). This was generated using data from the majority of patients and tested on a random subset in a split-reliability analysis. Significance was defined as p<0.05.

## Results

### Patient cohort

A total of 95 patients were identified, 78 of whom had serum samples of sufficient volume available; these comprised 38 with an infectious aetiology (17 HSV, 7 varicella zoster virus [VZV], 5 tuberculosis, 3 bacterial, 2 dual infection (1 TB and HIV; 1 cryptococcus and VZV), 1 influenza A, 1 measles, 1 HIV, 1 toxoplasmosis); 20 immune-mediated cases (9 antibody-mediated, 8 ADEM, 1 paraneoplastic, 1 vasculitis, 1 multiple sclerosis), and 20 of unknown aetiology. For 37 patients CSF samples of sufficient volume were available, 19 with an infectious aetiology (11 HSV, 6 VZV, 1 JC virus, and 1 toxoplasmosis), 9 immune-mediated (5 ADEM, 3 antibody-mediated, 1 paraneoplastic), and 8 of unknown aetiology. Additional samples were available to assess the MMP panel for 62 serum and 18 CSF samples.

### Comparison between intrathecal and vascular compartments

The following mediators were not identified in >80% of the cohort and were therefore removed from further analysis: TNFR1 and 2, eSelectin, CXCL9, IL-17α, and VEGFα.

**Fig 1:**
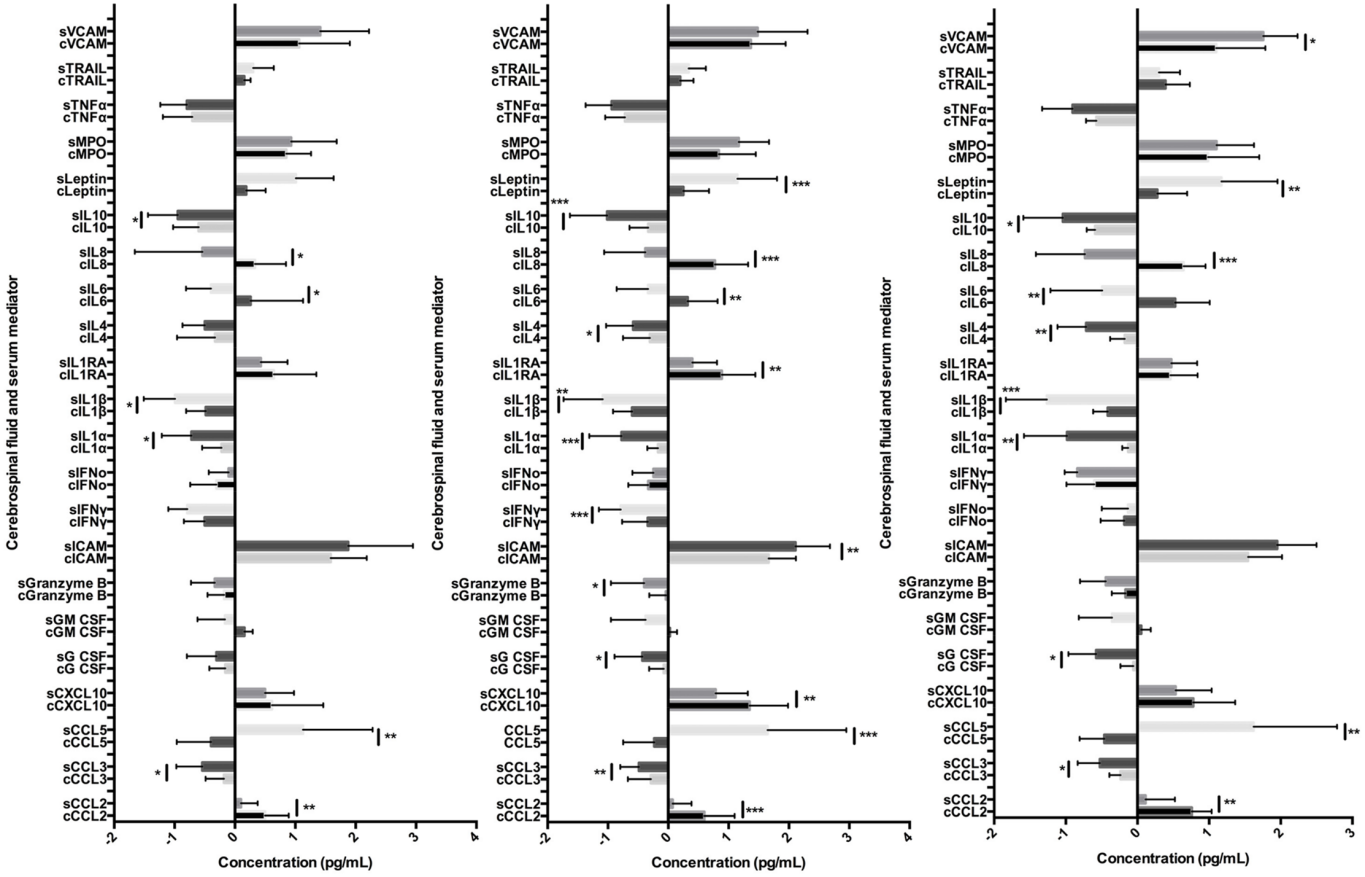
Concentrations of mediators in the CSF and serum of patients with encephalitis of immune-mediated, infectious and unknown aetiologies.

The mediators that tended to be found at higher concentrations in either the CSF or the serum were similar across all the aetiological groups (Fig 1). Significantly higher relative concentrations of several mediators, including IL-1α, IL-1β, IL-10, and CCL-2, were seen in the CSF than the serum for all aetiological groups. Also, for the infectious and unknown groups, higher concentrations of IL-4 and leptin occurred in the CSF than the serum. For the cohort overall the only mediators which showed a positive correlation between concentrations in the CSF and serum were MPO (tau b [95%CI] 0.42 [0.11–0.74], p = 0.009), CXCL-10 (0.43 [0.18–0.68], p = 0.01), and IL-4 (0.31 [0.01–0.61], p = 0.04).

### Univariate analysis of mediator profiles for different aetiological groups

Univariate analysis of mediator concentrations showed many similarities but also several differences between the aetiological groups (Tables 3 and 4). The predominant differences were between the infectious and immune-mediated groups and also between the unknown and the immune-mediated group. No significant differences were found between the infectious and unknown groups in either the CSF or the serum.

**Table 3:** Median concentrations of identified mediators between the aetiological groups of encephalitis in serum.

	Aetiological Group			P value		
Mediator	Immune-mediated	Infectious	Unknown	IMM vs INF	IMM vs UNK	INF vs UK
**CCL2**	0.041 [-0.560 to 0.652]	0.042 [-0.726 to 0.664]	0.040 [-0.791 to 1.041]	0.64	0.99	0.59
**CCL3**	-0.548 [-1.343 to 0]	-0.526 [-1.487 to -0.047]	-0.473 [-1.059 to -0.053]	0.62	0.99	0.71
**CCL5**	1.390 [-0.986 to 2.694]	2.129 [-0.849 to 2.966]	2.057 [-0.801 to 2.669]	0.15	0.50	0.35
**CXCL10**	0.400 [0.030 to 1.868]	0.728 [-0.038 to 1.778]	0.429 [-0.320 to 1.720]	0.17	0.83	0.08
**G CSF**	-0.153 [-1.291 to 0.771]	-0.283 [-1.151 to 0.451]	-0.710 [-1.112 to 0.054]	0.50	0.23	0.55
**GM CSF**	0.056 [-1.125 to 0.258]	-0.372 [-1.359 to 0.540]	-0.446 [-1.393 to 0.396]	0.10	0.42	0.26
**Granzyme B**	**-0.222 [-1/049 to 0.298]**	-0.187 [-1.141 to 0.064]	**-0.488 [-1.382 to 0.176]**	0.49	**0.05**	0.24
**IFNβ**	-0.747 [-2.552 to 0.061]	-0.213 [-2.502 to 0.126]	-0.897 [-2.427 to 0.277]	0.25	0.94	0.24
**IFNγ**	-0.814 [-1.660 to -0.349]	-0.860 [-1.601 to 0.180]	-0.914 [-1.158 to -0.467]	0.53	0.28	0.80
**IFNο**	-0.210 [-0.617 to 0.605]	-0.306 [-0.770 to 0.413]	-0.186 [-0.650 to 0.882]	0.36	0.99	0.37
**ICAM**	**1.694 [-0.302 to 4.401]**	**2.007 [0.297 to 2.919]**	1.976 [0.778 to 2.656]	**0.05**	0.18	0.72
**IL-1α**	-0.640 [-1.925 to 0.058]	-0.904 [-1.694 to 0.103]	-1.271 [-1.652 to 0.112]	0.46	0.28	0.86
**IL-1β**	-0.897 [-1.939 to -0.269]	-1.005 [-2.342 to -0.073]	-1.005 [-2.073 to -0.388]	0.39	0.27	0.89
**IL-1RA**	0.261 [-0.261 to 1.214]	0.159 [-0.107 to 1.609]	0.374 [-0.147 to 1.019]	0.94	0.30	0.15
**IL-4**	-0.321 [-1.387 to 0.007]	-0.625 [-1.332 to 0.192]	-0.812 [-1.274 to -0.161]	0.09	0.14	0.79
**IL-6**	-0.125 [-1.617 to -0.036]	0.070 [-1.469 to 0.421]	-0.152 [-1.717 to 1.246]	0.25	0.87	0.46
**IL-8**	-1.127 [-2.468 to 2.207]	-0.256 [-2.123 to 1.194]	-0.782 [-1.892 to 0.480]	0.10	0.23	0.48
**IL-10**	-0.845 [-2.105 to 0.046]	-0.957 [-2.446 to 0.160]	-1.051 [-2.334 to -0.110]	0.36	0.55	0.53
**Leptin**	0.911 [0.013 to 2.041]	1.178 [-0.189 to 2.414]	0.914 [0.102 to 2.625]	0.82	0.94	0.74
**MMP8**	0.001 [-2.289 to 1.262]	-0.100 [-2.089 to 1.464]	-0.020 [-2.300 to 1.561]	0.43	0.87	0.40
**MMP9**	0.854 [-2.357 to 1.728]	0.905 [-2.455 to 4.747]	1.248 [-2.790 to 2.557]	0.98	0.19	0.20
**MPO**	**0.687 [-0.301 to 2.276]**	**1.119 [-0.431 to 2.289]**	**1.026 [-0.040 to 2.158]**	**0.01**	**0.03**	0.80
**TNFα**	**-0.589 [-1.636 to -0.215]**	**-0.913 [-1.668 to -0.099]**	-0.993 [-1.610 to -0.138]	**0.04**	0.32	0.27
**TRAIL**	0.139 [-0.107 to 1.139]	0.274 [0 to 1.301]	0.224 [-0.077 to 1.210]	0.23	0.51	0.44
**VCAM**	1.728 [-0.435 to 2.383]	1.600 [-0.475 to 2.551]	1.854 [0.675 to 2.519]	0.85	0.11	0.19

Abbreviations: IMM Immune-mediated; INF Infectious; UNK Unknown aetiology. Values are median-centred as described and expressed as pg/mL.

**Table 4:** Median concentrations of identified mediators between the aetiological groups of encephalitis in cerebrospinal fluid.

	Aetiological Group			p value		
Mediator	Immune-mediated	Infectious	Unknown	IMM vs INF	IMM vs UNK	INF vs UK
**CCL2**	0.250 [-0.176 to 0.783]	0.459 [-0.755 to 0.683]	0.656 [-0.470 to 1.095]	0.41	0.83	0.13
**CCL3**	**-0.289 [-0.407 to -0.136]**	-0.383 [-0.892 to -0.102]	**-0.413 [-0.660 to -0.236]**	0.36	**0.03**	0.35
**CCL5**	-0.625 [-0.986 to 0.954]	-0.439 [-1.144 to -0.165]	-0.573 [-1.107 to 0.685]	0.61	0.64	0.66
**CXCL10**	0.242 [-0.50 to 1.729]	1.203 [-0.060 to 1.686]	0.986 [-0.068 to 1.561]	0.22	0.56	0.66
**G CSF**	0.148 [-0.269 to 2.360]	0.275 [-0.273 to 2.218]	-0.091 [-0.423 to 2.091]	0.97	0.30	0.66
**GM CSF**	**0.116 [-0.210 to 0.345]**	**-0.059–0.627 to 0.011]**	-0.153 [-0.423 to 0.146]	**0.01**	0.28	0.66
**Granzyme B**	-0.351 [-0.381 to 1.839]	0.216 [-0.495 to 1.620]	-0.223 [-0.489 to 1.570]	0.55	0.91	0.56
**IFNβ**	0.202 [-0.167 to1.917]	1.935 [-0.427 to 2.266]	1.968 [-0.407 to 2.138]	0.22	0.11	0.37
**IFNγ**	-0.857 [-1.042 to -0.590]	-0.595 [-1.265 to 0.981]	-0.721 [-1.317 to 1.185]	0.17	0.49	0.61
**IFNο**	0.109 [-0.697 to 0.289]	-0.380 [-0.510 to 1.053]	-0.476 [-0.785 to 1.256]	0.72	0.73	0.35
**ICAM**	1.465 [0.786 to 2.272]	1.437 [0.622 to 2.118]	1.243 [0.8502 to 2.003]	0.91	0.64	0.89
**IL-1α**	-0.145 [-0.384 to 0.058]	-0.211 [-1.046 to 0.015]	-0.216 [-0.793 to -0.117]	0.72	0.17	0.13
**IL-1β**	**-0.452 [-0.581 to -0.245]**	-0.600 [-1.005 to -0.307]	**-0.602 [-0.801 to -0.467]**	0.08	**0.01**	0.56
**IL-1RA**	**0.097 [-0.010 to 0.158]**	**0.536 [-0.708 to 1.609]**	0.134 [-0.504 to 1.096]	**0.01**	0.56	0.11
**IL-4**	**-0.127 [-0.393 to 0.088]**	**-0.291 [0.708 to -0.132]**	**-0.339 [-0.569 to -0.247]**	**0.03**	**0.02**	0.47
**IL-6**	-0.133 [-0.498 to 2.066]	0.661 [-0.334 to 1.924]	0.778 [0.001 to 1.797]	0.36	0.17	0.06
**IL-8**	**-0.122 [-0.418 to 0.156]**	**0.318 [-0.553 to 1.319]**	**0.436 [-0.329 to 1.102]**	**0.04**	**0.03**	0.61
**IL-10**	**-0.508 [-0.855 to -0.384]**	-0.606 [-1.448 to 0.007]	**-0.721 [-1.244 to -0.569]**	0.72	**0.02**	0.15
**Leptin**	0.653 [0.402 to 2.486]	1.960 [-0.007 to 2.344]	2.045 [0.407 to 2.216]	0.78	0.49	0.61
**MMP8**	0.300 [-3.423 to 1.581]	-0.132 [-2.974 to 1.418]	1.198 [-3.276 to 4.094]	0.97	0.17	0.43
**MMP9**	1.031 [-4.132 to 2.484]	-0.024 [-4.549 to 4.268]	1.207 [-3.374 to 2.873]	0.91	0.56	0.39
**MPO**	**0.560 [0.210 to 0.927]**	**1.030 [0.060 to 1.944]**	1.034 0.142 to 2.147]	**0.01**	0.30	0.47
**TNFα**	-0.465 [-0.114 to -0.825]	-0.588 [-1.574 to -0.131]	-0.746 [-1.371 to -0.459]	0.25	0.11	0.52
**TRAIL**	0.046 [-0.155 to 0.298]]	0.139 [-0.339 to 1.537]	0.284 [-0.293 to 1.741]	0.17	0.06	0.17
**VCAM**	1.113 [-0.561 to 1.905]	1.121 [-1.170 to 2.192]	0.896 [-0.922 to 1.930]	0.84	0.54	0.83

Abbreviations: IMM Immune-mediated; INF Infectious; UNK Unknown aetiology. Values are median-centred as described and expressed as pg/mL.

**Fig 2:**
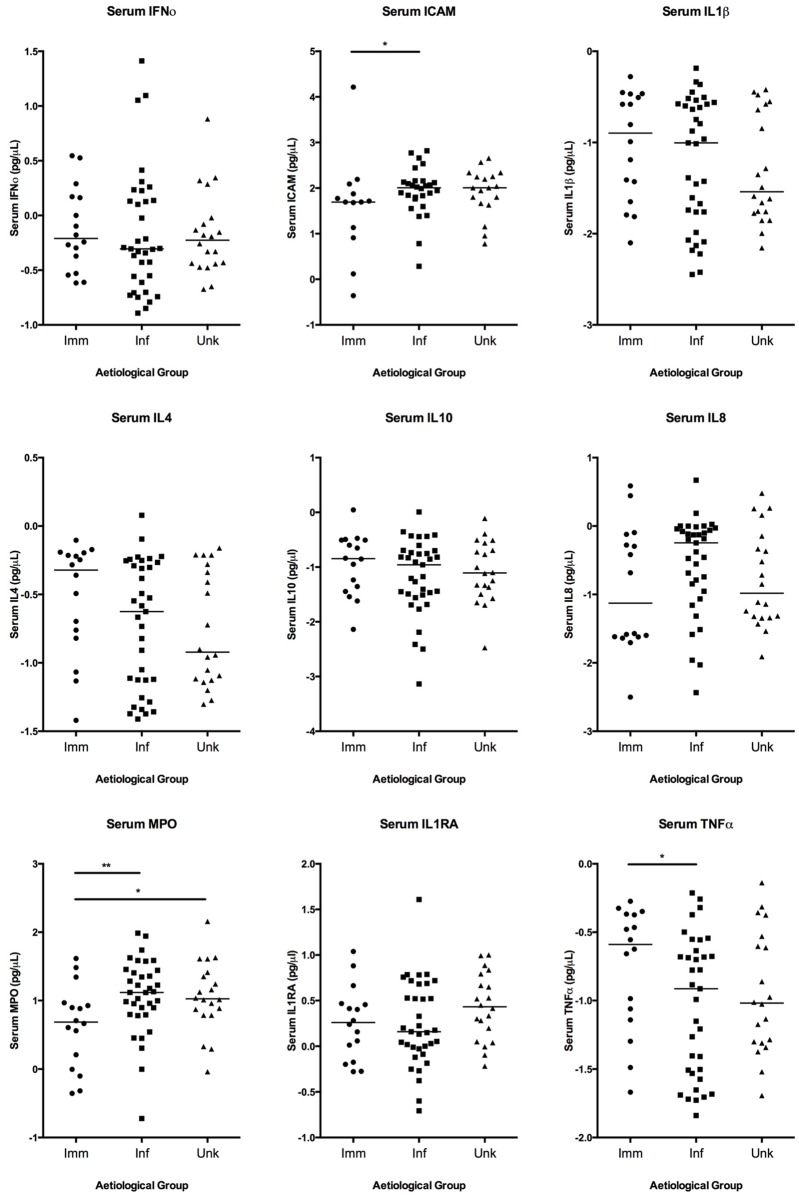
Serum concentrations of mediators in patients with encephalitis of immune-mediated, infectious and unknown aetiology. Bars represent median concentration.

**Fig 3:**
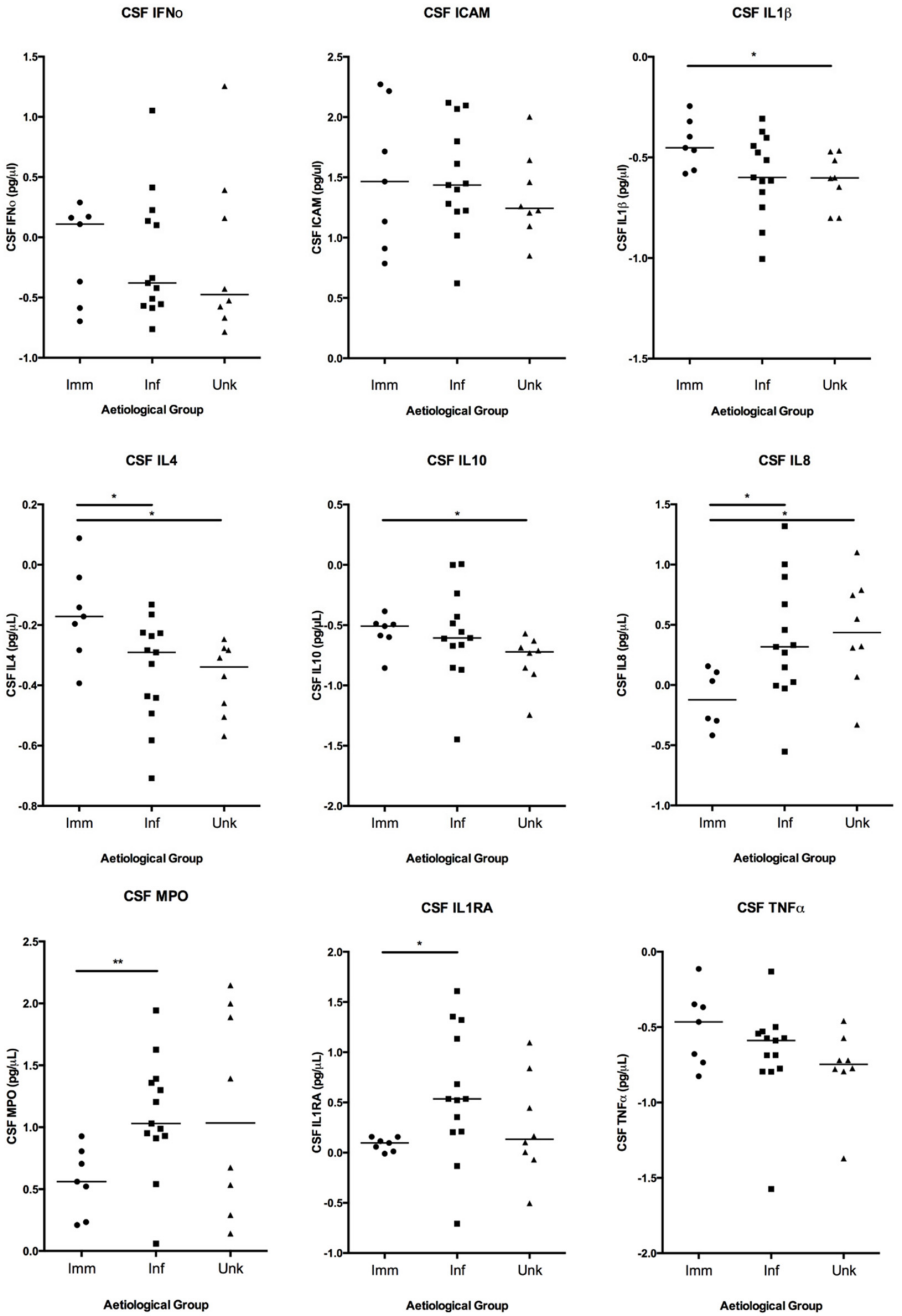
CSF concentrations of mediators identified in patients with encephalitis of immune-mediated, infectious and unknown aetiology.

In particular, higher concentrations of MPO were identified in both the serum and CSF of patients with infectious than immune-mediated aetiologies (p = 0.01 and p = 0.006 respectively) (Figs 2 and 3). Interestingly, the serum MPO concentration was also higher in those with unknown aetiology than immune-mediated disease (p = 0.03). In addition, median CSF concentrations for several of the mediators were similar for the infectious and unknown groups, but differed for the immune-mediated group: specifically, higher median concentrations of IL-8 were seen in both infectious and unknown cases as opposed to those that were immune-mediated; in contrast higher median concentrations of IL-4 were found in those with immune-mediated disease than either of the other groups. To assess whether the predominant infectious aetiology, which was the HSV group, had a similar mediator pattern to that seen in the rest of the infectious group the two were compared (Table 5). There were no statistically significant differences for any of the mediators assessed in CSF between those with HSV and the rest of the group with an infectious aetiology. In serum there were also no significant differences between the two groups other than for IL-1α and ICAM. However, IL-1α did not differ significantly different between infectious and other aetiological groups overall, although ICAM approached significance.

**Table 5:** Comparison between the median mediator concentrations in serum and CSF in patients with HSV encephalitis and the rest of the group with encephalitis of an infectious aetiology.

Serum				Cerebrospinal fluid		
Mediator	HSV (n = 17)	Non-HSV Infection (n = 21)	p value	HSV (n = 11)	Non-HSV Infection (n = 7)	p value
**CCL2**	0.100 [-0.646 to 0.412]	0.053 [-0.244 to 0.664]	0.65	0.413 [0.086 to 0.683]	0.243 [-0.755 to 0.664]	0.48
**CCL3**	-0.401 [-0.906 to -0.047]	-0.561 [-0.688 to -0.072]	0.12	-0.345 [-0.537 to -0.130]	-0.455 [-0.892 to -0.102]	0.43
**CCL5**	1.111 [-0.845 to 2.789]	2.037 [-0.849 to 2.966]	0.1	-0.364 [-1.144 to 0.453]	-0.359 [-0.849 to 0.481]	0.98
**CXCL10**	0.801 [-0.38 to 1.723]	0.772 [0.095 to 1.778]	0.9	1.118 [-0.60 to 1.686]	1.240 [1.104 to 1.465]	0.65
**G CSF**	-0.302 [-1.100 to 0.451]	-0.526 [-1.126 to 0.135]	0.16	0.815 [-0.096 to 2.141]	0.813 [-0.273 to 2.218]	0.10
**GM CSF**	-0.247 [-1.145 to 0.540]	-0.484 [-1.263 to 0.359]	0.23	-0.065 [-0.272 to 2.218]	-0.168 [-0.627 to 0.079]	0.37
**Granzyme B**	-0.350 [-1.152 to 0.857]	-0.437 [-1.821 to 0.310]	0.65	0.524 [-0.857 to 1.620]	0.117 [-0.495 to 1.366]	0.35
**ICAM**	**1.870 [0.397 to 2.804]**	**2.305 [1.645 to 2.919]**	**0.03**	1.402 [0.622 to 2.118]	1.625 [1.216 to 2.097	0.40
**IFNα**	-0.159 [-0.313 to 0]	-0.291 [-1.151 to 0.223]	0.64	0.013 [-1.128 to 1.837]	0.549 [-0.831 to 2.090]	0.39
**IFNβ**	-0.562 [-1.892 to 0]	-1.103 [-2.502 to 0.126]	0.12	1.441 [-0.364 to 2.117]	1.104 [-0.427 to 2.266]	0.62
**IFNγ**	-0.153 [-0.695 to 0.413]	-0.313 [-0.770 to 0.308]	0.18	-0.522 [-0.960 to 0.041]	-0.249 [-1.265 to 0.981]	0.48
**IFNο**	-0.824 [1.561 to -0.269]	-0.760 [-1.601 to 0.180]	0.6	-0.340 [-0.569 to 0.413]	0.105 [-0.510 to 1.053]	0.14
**IL-1α**	**-0.584 [-1.596 to 0.103]**	**-0.933 [-1.694 to -0.229]**	**0.04**	-0.226 [-0.632 to 0.015]	-0.280 [-1.046 to 0.005]	0.77
**IL-1β**	-0.894 [-2.135 to -0.073]	-1.241 [-2.342 to -0.270]	0.1	-0.559 [-0.748 to -0.402]	-0.635 [-1.005 to -0.307]	0.53
**IL-1RA**	0.366 [-0.107 to 1.609]	0.425 [-0.092 to 0.985]	0.67	0.741 [-0.133 to 1.609]	0.339 [-0.708 to 1.321]	0.29
**IL-4**	-0.447 [-1.320 to 0.192]	-0.692 [-1.332 to 0.038]	0.1	-0.320 [-0.493 to -0.165]	-0.398 [-708 to -0.132]	0.45
**IL-6**	-0.115 [-0.728 to 0.254]	-0.468 [-1.212 to 0.421]	0.11	0.723 [-0.068 to 1.847]	0.767 [-0.334 to 1.924]	0.93
**IL-8**	-0.335 [-2.123 to 1.194]	-0.417 [1.433 to 0.186]	0.7	0.507 [-0.027 to 1.319]	0.160 [-0.553 to 1.003]	0.24
**IL-10**	-0.956 [-2.446 to 0.007]	-1.062 [-2.408 to 0.160]	0.6	-0.428 [-0.870 to 0.007]	-0.801 [-1.448 to -0.485]	0.09
**Leptin**	1.135 [-0.007 to 1.994]	1.154 [-0.189 to 2.414]	0.9	1.366 [-0.007 to 2.267]	1.361 [0.040 to 2.344]	0.99
**MMP8**	-0.398 [-2.089 to 0.754]	-0.018 [-1.669 tp 1.464]	0.18	0.135 [-1.662 to 1.418]	-1.450 [-2.974 to 0.626]	0.07
**MMP9**	1.124 [-2.455 to 4.747]	0.721 [-1.076 to 4.268]	0.45	-0.186 [-4.295 to 3.936]	0.111 [-4.549 to 4.268]	0.87
**MPO**	1.222 [0.305 to 2.216]	1.130 [-0.431 to 2.289]	0.6	1.032 [0.060 to 1.627]	1.196 [0.540 to 1.944]	0.57
**TNFα**	-0.854 [-1.668 to -0.099]	-1.006 [-1.609 to -0.320]	0.29	-0.650 [-0.795 to -0.499]	-0.710 [-1.574 to -0.131]	0.76
**TRAIL**	0.358 [0.015 to 0.730]	0.323 [0 to 1.301]	0.7	0.096 [-0.339 to 0.493]	0.417 [0.088 to 1.537]	0.22
**VCAM**	1.291 [-0.435 to 2.303]	1.634 [-0.475 to 2.551]	0.2	1.189 [0.555 to 2.192]	0.956 [-1.170 to 2.092]	0.65

Values are median-centred as described and expressed as median [range] in pg/mL.

### Heatmaps of mediator profiles for different aetiological groups

**Fig 4:**
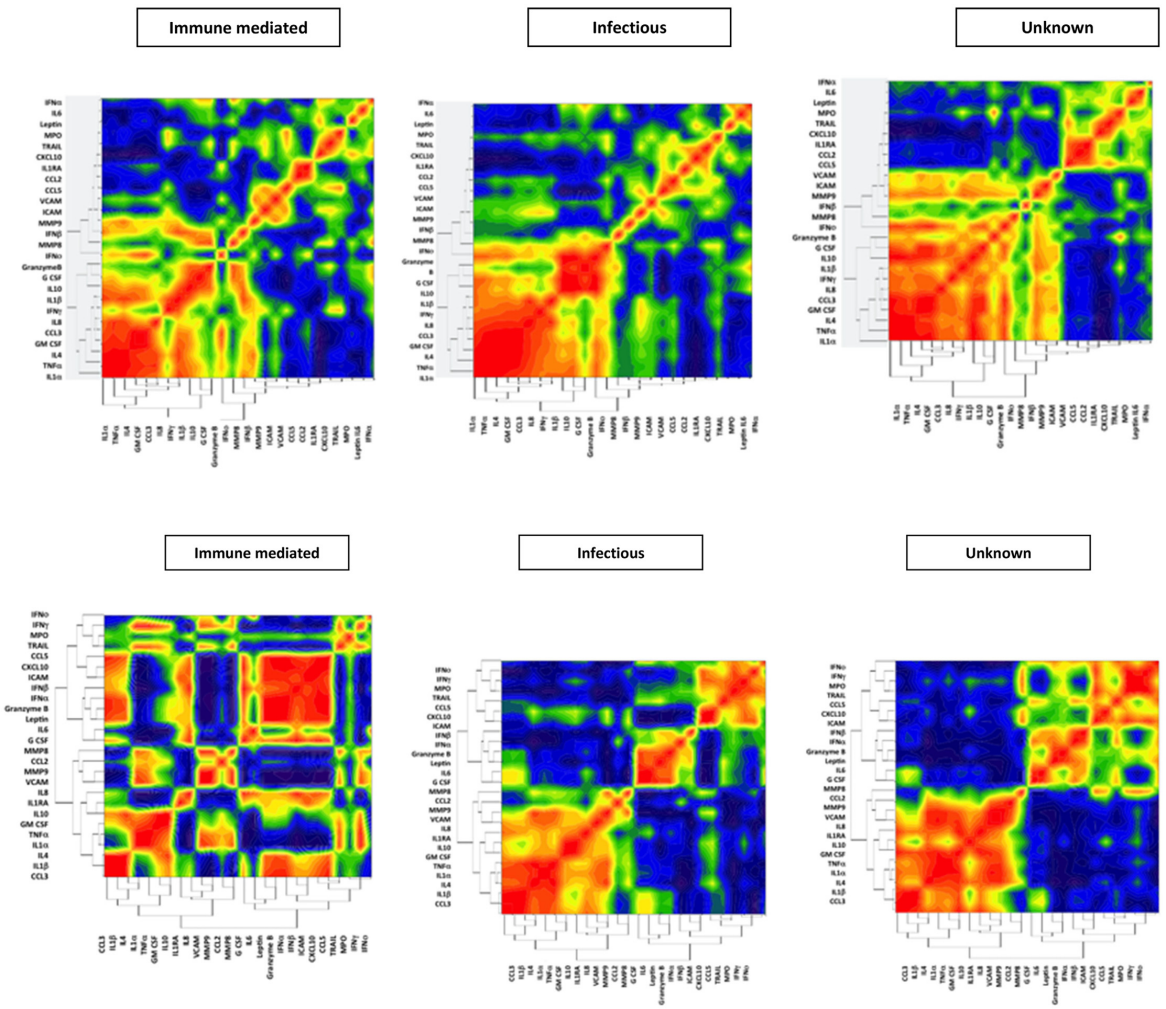
The heatmaps give a visual representation of how closely concentrations of different mediators correlate in the samples by nearest neighbour correlation. Using a hierarchical cluster analysis the mediators are listed in the same order for each of the three aetiological groups to allow a visual comparison of the pattern between the groups. In the serum (a) the pattern of mediator correlations is similar for all three aetiological groups, where-as in the cerebrospinal fluid (b) the pattern differed between immune-mediated and infectious aetiologies, and the pattern for those of unknown aetiology is closer to that seen with infection.

Hierarchical cluster analysis of mediators from all patients identified two broad clusters of mediators, which were used in the heat-maps. The first cluster of serum mediators contained IL-1α, IL-1β and IL-10, in addition to other mediators; in CSF these same mediators were clustered together with the addition of IL-1RA. The second cluster in serum contained several mediators including IFNα, IFNβ, IFNγ and IFNο; these same mediators clustered together in CSF. Heat-maps derived from serum mediator concentrations showed strong positive correlations between mediators in the first cluster and strong negative correlation in between mediators in the second cluster in all aetiological groups and this did not appear to be substantially different between groups (Fig 4). However, heat-maps derived from CSF mediator concentrations appeared to demonstrate strong positive correlations between the first cluster of mediators in the infectious group that was not present in those with immune-mediated aetiology. To assess whether the mediator heat-map from the unknown group better reflected the infectious or immune-mediated group these were compared. The heat-maps of CSF mediators appeared to be similar for the infectious and unknown groups and both appeared substantially different from those with an immune-mediated aetiology.

### Discriminant function analysis of mediator profiles for different aetiological groups

To see which specific mediators might be used to distinguish between aetiological groups we used discriminant function analysis for pairwise comparisons of the groups. For discriminant function analysis of serum data this generated a model that used serum MPO alone to distinguish 31 (89%) of 35 samples as infective and five (31%) of 16 as immune-mediated (Wilks’ lambda 0.83, p = 0.008). This technique also generated a model using serum MPO alone to distinguish 17 (85%) of 20 samples of unknown aetiology and six (38%) of 16 immune-mediated samples (0.78, p = 0.009); both were confirmed on the leave-one-out cross validation. No serum variables qualified as distinguishing samples of infective versus unknown aetiology. A serum MPO concentration cut-off of >0.8pg/mL correctly identified 27 (77%) infective, 17 (85%) as unknown, and 8 (50%) immune-mediated cases; to assess the usefulness of this cut off value for MPO this was then applied to samples from 7 patients (3 infective and 4 immune-mediated) that had not been included in the initial analysis. In this split-reliability analysis all three cases were identified as infective but none of the four were identified as immune-mediated.

Discriminant function analysis of CSF data generated a model which again used MPO alone to correctly classify 11 (85%) of 13 cases as infective and four (67%) of six cases as immune-mediated (0.77, p = 0.036); this was confirmed on the leave-one-out cross validation. A CSF MPO concentration cut-off of 1.0pg/mL correctly classified nine (69%) infective cases and four (67%) immune-mediated cases. The proportion of cases correctly classified was the same when applied to the raw data using a cut-off of 1000pg/mL. To assess the usefulness of this cut off value we assessed this in 9 patients (6 infective and 3 immune mediated) not included in the original analysis. In this split-reliability analysis three of six and one of three cases were classified as infective or immune-mediated respectively.

**Fig 5:**
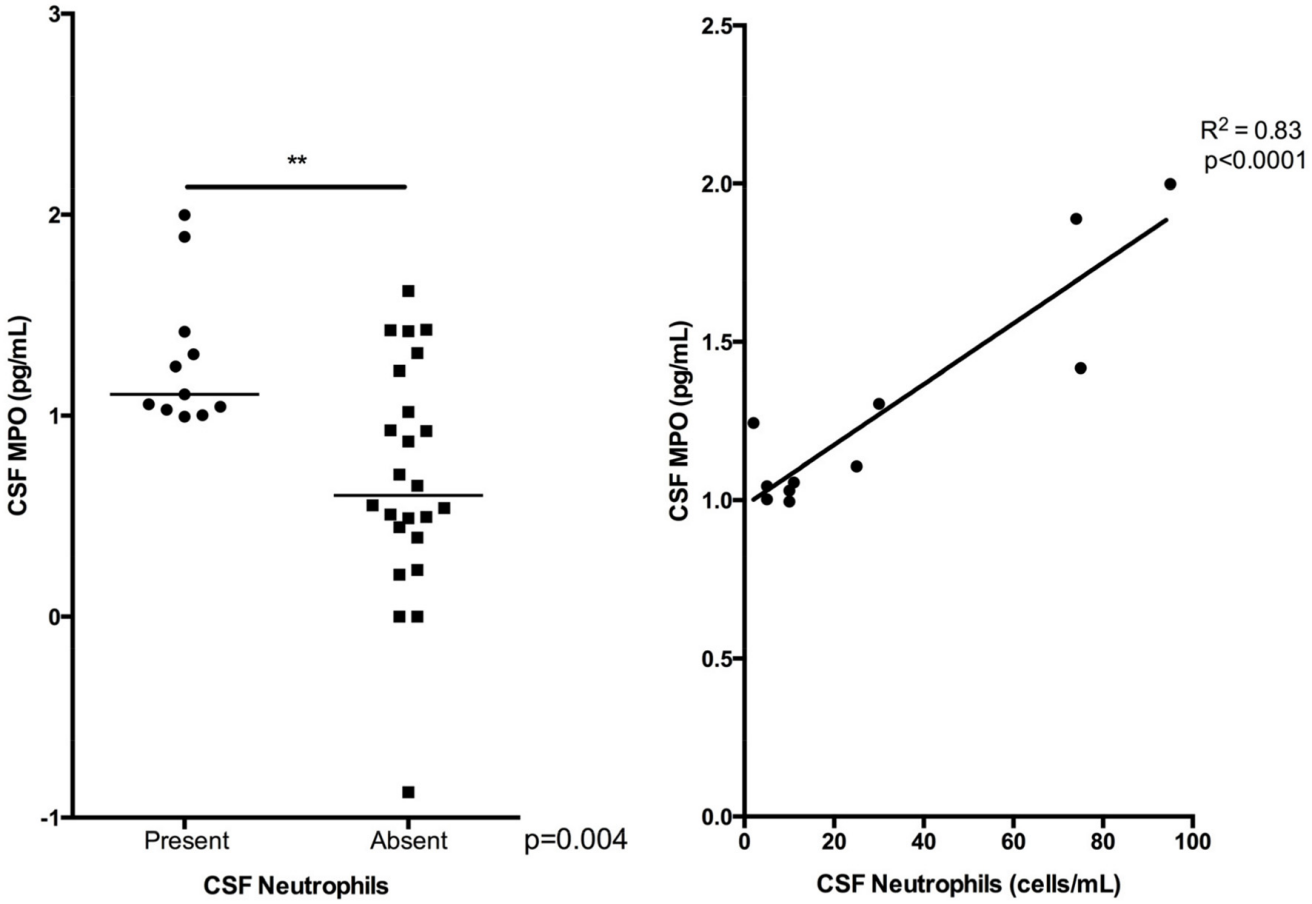
Cerebrospinal fluid myeloperoxidase concentration and cerebrospinal fluid neutrophil count for all cases of encephalitis. Because at low levels neutrophils are just recorded as present or absent, and at higher levels actual counts are given we looked at CSF MPO concentrations using both these parameters. CSF neutrophil counts were given for 11 patients.

As MPO is released during degranulation of neutrophils, we assessed whether these findings may reflect the relative neutrophil concentration between the different aetiological groups. Eight (62%) of the infective group had detectable neutrophils in the CSF, as opposed to only one (14%) of the immune-mediated group (p = 0.004). Moreover, across the whole cohort there was a strong positive correlation between CSF neutrophil count and CSF MPO concentration (tau b [95% CI] 0.70 [0.31–1], p<0.0001) (Fig 5). In the split reliability analysis neutrophils were detectable in the CSF for three of five infective samples and none of two immune-mediated samples, for whom data were available. There were no significant differences in the proportion of CSF samples with a detectable neutrophil count between the other aetiological groups. There was no significant correlation between either the CSF or the serum concentration of MPO and time from symptom-onset to sample collection. Discriminant analysis of CSF mediators identified eight (100%) of the unknown and five (83%) of immune-mediated samples using IL-8 and CCL-3 (Wilks’ lambda 0.62, p = 0.018 and 0.21, p<0.0001 respectively). Discriminant function analysis was again not able to generate a model that could distinguish CSF samples of an infective aetiology from samples of an unknown aetiology.

## Discussion

This study has shown that the cytokine and associated mediator profiles in UK patients with infectious encephalitis differ from those with immune-mediated aetiologies, particularly in the CSF. For patients with unknown aetiology patterns in CSF most closely resembled infectious cases, suggesting they may have undiagnosed infection. MPO was the mediator of greatest significance, with higher concentrations in patients with infection, and CSF MPO concentration was strongly correlated with CSF neutrophil count.

There are an estimated 0.07–12.6 cases of encephalitis per 100,000/year [1,2,10,26]. The most common cause is infection and immune-mediated cases are being recognised increasingly [2,27]. However, the aetiology remains unknown in 15–60% of cases, despite comprehensive investigation [2,10,28]. There is mounting evidence that the host inflammatory process may play an important role in the pathogenesis whatever the underlying aetiology [11–16]. These mediators modulate both the innate and adaptive responses and regulate leucocyte infiltration [29,30]. However, little is known about how the responses compare between encephalitis of different causes.

Therefore, we analysed CSF and serum samples from 95 patients with encephalitis recruited prospectively in the HPA Encephalitis Study [2]. We found that there were many similarities across the aetiological groups. Particularly, mediators that were identified at higher concentrations in the CSF than in the serum were similar between all aetiological groups; also those present at higher concentrations in the serum were similar across all aetiological groups. In line with a previous study of viral encephalitis we found higher relative levels of CCL-2 and IL-6 in the CSF than serum and higher levels of CCL-5 in serum; in our study this was also found in those with immune-mediated and unknown aetiologies [11]. However, several differences between mediators, particularly in the CSF, were identified for the different aetiological groups. Cluster analysis identified a group of mediators containing IL-1α, IL-1β, IL-1RA and IL-10, which are all strongly associated with inflammation, whatever the cause [31]. CSF heat-maps demonstrated that the pattern of mediator response for those with encephalitis of unknown cause was similar to that for infectious rather than immune-mediated causes, suggesting they may actually have infectious encephalitis, but the causative agent has not been identified.

Discriminant analysis of both CSF and serum identified MPO as the mediator that most strongly discriminated cases of an infective or unknown aetiology from immune-mediated cases. CSF concentrations of MPO correlated with CSF neutrophil count, suggesting that CSF neutrophils may be the primary source of MPO identified. A higher percentage of patients had detectable neutrophils in the CSF in the infectious as opposed to immune-mediated group, although this was not as strong an association as the MPO concentration, perhaps suggesting that the MPO in the CSF may also reflect neutrophil extracellular traps [32]; these are networks of extracellular fibres produced by neutrophils, made up of DNA, MPO, and other proteins, that bind microorganisms. Or the MPO detected may in part reflect neutrophils that were previously present in CSF and have migrated into the brain parenchyma or back into serum.

Neutrophils are known to be an important part of the host response early in viral encephalitis, before lymphocytes predominate; in bacterial central nervous system infections the neutrophil counts predominate, and are typically much higher. Interestingly, neutrophils have been identified recently in murine models of HSV encephalitis, and may be important for reducing viral load, albeit whilst also being associated with the production of, potentially neurotoxic, reactive oxygen species [33–35]. Histopathological descriptions of clinical HSV encephalitis have reported a predominance of neutrophils in the first 2–3 days [22]. Although after 10–15 days macrophages and lymphocytes are the predominant populations, the progressive necrosis leads to further neutrophil infiltration, which would be supported by our findings [22]. Histopathological data for immune-mediated encephalitis are limited, but small series describe either no leucocyte infiltration or scanty lymphocytes without neutrophils, and in a subset cytotoxic T lymphocytes may predominate [16,36–38]. Similar observations have been made in rodent models [37].

Our study suggests that MPO in the CSF could be explored as a biomarker for a potential infectious aetiology in patients with encephalitis. Also serum MPO was also associated with an infective aetiology so may represent a potential biomarker and both require validation in prospective studies. For example interesting developments have been made in point-of-care tests for protein biomarkers, such as the lateral flow assays developed for CSF lactate to distinguish bacterial from viral meningitis [39]. In addition to MPO significant differences were found in serum for two other mediators between the groups. Interestingly TNFα was identified at higher concentrations in the immune-mediated than infectious group. Although TNFα has a broad spectrum of activity, this finding may perhaps reflect the key role it has in promoting the proliferation and differentiation of B cells, which is a pivotal process in the development of autoantibodies [40–43]. ICAM also approached significance, being present at greater concentration in the infectious than immune-mediated group, potentially as a corollary of greater blood-brain barrier permeability as would be expected given the greater pleocytosis that is typically seen in infectious cases [1, 44–46].

In CSF there were many more differences in the concentrations of mediators between the aetiological groups. Specifically, higher concentrations of IL-8 were identified in both the infectious and unknown group as opposed to immune-mediated cases, which would be consistent with the key finding of the role of neutrophils in our study, given the important role of IL-8 in neutrophil activation [47–49]. In addition, greater concentrations of IL-1RA, IL-4, IL-10 and IL-1β were found in the immune-mediated group. That both anti-inflammatory (IL-1RA, IL-4 and IL-10) and pro-inflammatory (IL-1β) mediators were elevated in the same aetiological group support the complex regulatory interaction between these mediators and the importance of assessing the broad cytokine/chemokine milieu [11].

Interestingly no significant differences were found in the concentration of any of the mediators between the infectious and unknown groups in either the serum or the CSF. Only one other study has assessed profiles between encephalitis aetiologies and identified a higher concentration of IFNγ and TNFR1 in 13 patients with HSV than 15 with ‘non-herpetic limbic encephalitis’ [12]. We did not identify any difference in the relative concentration of interferons between infectious cases and those of a proven immune-mediated aetiology; although it is unclear how this relates to the heterogeneous group of ‘non-herpetic limbic encephalitis’ in the earlier study. That the interferon response, which has been associated with a greater risk of developing HSV encephalitis, was not found to be significant in our study is a potentially interesting negative, although it may be a consequence of the number of CSF samples available or the pooling of infectious aetiologies [40,50]. Also some mediators, which have been identified in previous studies, such as VEGFα, were not detected in significant concentrations in the majority of patients in this study [51]. Although this may be because previous studies have focused on pathogens associated with a viraemia and marked blood-brain barrier damage, rather than primary neurotropic infection, as is seen in HSV, which was the majority of infectious cases in our study [14, 51].

Pooling the patients with different infectious aetiologies together, allowed analysis of factors which were common between them, and which distinguished them from immune-mediated encephalitis. It also allowed a comparison with those whose aetiology was unknown., This analysis makes the assumption that the pathology is similar in infectious cases. Whilst most is known about HSV infection, which is associated with necrosis, neutrophil invasion, haemorrhagic change and secondary autoimmunity in some, fewer histopathological data are available for other aetiologies [22,52,53]. Indeed focusing on a single infectious cause would have reduced the numbers of patients, and may have led to us overlooking similarities with those whose aetiology was unknown. To try to address whether grouping the infectious samples together, when the majority were due to HSV, had resulted in skewing the results of the infectious group, we looked for differences between the HSV and the other infectious aetiologies. We found that the concentration of all the mediators assessed in CSF was similar between the both the HSV and other infectious aetiologies. In serum IL-1α was present at higher concentrations in those with HSV than other infectious aetiologies but IL-1α did not differ significantly between the infectious group overall and the other aetiologies. Also in serum, ICAM was identified at lower concentrations in the HSV group than the other infectious aetiologies. This may be of importance as ICAM was found at a higher concentration in the infectious group overall than the immune-mediated group and this approached statistical significance. However, for the majority of mediators in serum and all the mediators in the CSF there were no significance differences between HSV and the rest of those of an infectious aetiology. This suggests that, although there are may be histopathological differences between the various infectious cases of encephalitis, there may be some similarities that support grouping the serum, and particularly CSF, host inflammatory response to maximise numbers when making comparisons with primarily immune-mediated cases. Although, this finding may reflect the relatively smaller numbers of samples available for this sub-group analysis and further studies are needed to assess the relative differences between specific pathogens in larger cohorts.

Although the patients for whom no aetiology could be identified had a mediator profile suggestive of infection, they had all undergone an extensive work up for infectious causes, through the HPA study [2,54]. There could be a number of explanations for failure to identify a pathogen. As most diagnoses are now based on CSF PCR for known causes, it may be that the pathogen was not present in CSF during the sampling time window, or that the specific pathogen was not looked for. In this cohort a subsequent analysis reported that there was no significant difference in the time from admission to first LP between those of known and unknown aetiology and also there was no difference between the time to first positive CSF or to first LP between these groups [54]. However, this did highlight that CSF samples tested for antibody were more likely to be positive if taken on days 14–28 as opposed to <6days. Indeed, previous studies have reported that the proportion of CSF positive by PCR is greatest early in admission, particularly after antiviral treatment [55,56]. Therefore, there may be a critical window for CSF testing dependent on both the pathogen and the laboratory investigation. Although, pathogen discovery approaches have identified novel causes of viral encephalitis [28], A subset of CSF from the HPA study have previously undergone one method of next-generation sequencing and this did not identify any novel pathogen nucleic acid [54]. It may be that the mediator profile could assist in identifying samples that may be more likely to have an infectious cause to undergo pathogen discovery. Alternatively the cause may reflect a para- or post-infectious inflammatory response provoked by peripheral infection, such as with influenza H1N1 [57].

In summary, whilst there were many similarities identified, the cytokine and chemokine profiles in the CSF of patients with encephalitis of an infectious aetiology differed from those with an immune-mediated aetiology; those in whom no cause could be identified best reflected those of an infectious aetiology. MPO may represent a potential biomarker to distinguish encephalitis due to infection versus immune-mediated causes, which may in part relate to neutrophils in the CSF.

### Funding and Acknowledgments

BDM is an NIHR Academic Clinical Lecturer and this work received support as part of an NIHR Doctoral Research Fellowship (NIHR-DRF-2010-03-97). TS received support from the NIHR Health Protection Research Unit in Emerging and Zoonotic Infections at Liverpool. TS was also supported by the MRC. The funders had no role in study design, data collection and analysis, decision to publish, or preparation of the manuscript. This work was supported by the laboratory team at the Vaccine Evaluation Unit, Public Health England, Manchester, UK. The views expressed are those of the authors and not necessarily those of the NHS, the NIHR, the Department of Health or Public Health England. The samples and data from these patients have been used in associated studies arising from the original Health Protection Agency (now Public Health England) Aetiological Study of Encephalitis in England.

## Supporting Information

S1 Table Supporting information sera. (PDF)

S2 Table Supporting information cerebrospinal fluid. (PDF)
