# Supplementary material for: Characteristic Cytokine and Chemokine Profiles in Encephalitis of Infectious, Immune-Mediated, and Unknown Aetiology
Source: PLoS One. 2016 Jan 25;11(1):e0146288. doi: 10.1371/journal.pone.0146288 (PMC4726626; doi:10.1371/journal.pone.0146288)
Supplement: S1 Table — (PDF) [file pone.0146288.s001.pdf]

Aetiology Group	G CSF	GM CSF	Granzyme B	IFNα2	IFNβ	IFNο
IMM	0.01414646	0.07729735	-0.0141465	1.82897507	2.00533218	-0.2426401
IMM	0.02226185	0.16697553	0.06628612	3.17744104	-1.5346589	0.54618954
IMM	-0.1385373	0.18155278	-0.3747048	-0.0581839	-0.0581839	0.28907156
IMM	0.77147806	0.07665732	-0.3814009	-0.1410355	-0.1674841	0.17096119
IMM	-0.0548979	0.25833061	-0.3507883	-0.0123653	-0.0664998	0.16204852
IMM	2.27971527	0.03563115	1.75888661	2.15085143	2.32720855	-0.6109025
IMM	-0.1669829	0.09796714	-0.0924401	-0.4763473	-0.1366018	-0.2688091
IMM	-0.003084	0.17341797	0.04704923	0.06013877	0.0528039	-0.0981683
IMM	-0.9186135	-1.1617599	-0.8532159	2.38325003	-2.1564865	-0.5295093
IMM	-0.4776355	-0.3989918	-0.3751216	2.79291022	-2.5560118	-0.1780179
IMM	-0.743964	-0.7111302	-0.5216607	2.80104588	-2.1403908	-0.3711419
IMM	-0.8649465	-0.5056155	-0.4641573	2.54059088	-1.6519839	-0.2964478
IMM	-1.0225995	-0.7116075	0.29769605	0.45745908	-1.4120095	-0.6169271
IMM	-0.8463675	-0.1011775	0.09489401	-0.4703294	-0.4241976	0
IMM	-1.3234731	-0.9994231	-0.837683	2.28420971	-1.0695648	-0.545176
IMM	-0.0568049	0.12689619	0.10620502	3.22269567	-1.408371	0.5263423
INF	1.92966827	-0.5845597	1.40883961	1.80080444	1.97716155	1.09538408
INF	0.3375539	0.42683817	0.20965342	1.99675928	-0.2904967	0.13751191
INF	-0.2066482	-0.0344098	-0.3429159	1.78462087	1.96097799	-0.557238
INF	-0.1720732	0.0380767	0.00149092	1.85710735	-0.3431953	-0.2145078
INF	-0.5211186	-1.0787047	0.18634522	2.83706999	-2.511852	-0.0239284
INF	-1.2436259	-1.5026398	-1.1616758	2.41313492	2.74470325	-0.7286616
INF	-1.2033061	-0.9611829	-1.1542912	2.31381717	-1.9440244	-0.7473031
INF	-0.9500669	-0.9922418	-0.7059138	2.46211805	-1.9272326	-0.3095675
INF	-0.6825104	-0.934055	-0.4739671	2.77804485	-1.1503713	-0.2357537
INF	-1.1797737	-1.4573226	-1.262995	2.31181578	2.6433841	-0.7019828
INF	-0.6708183	-0.7635723	-0.6803636	2.85564505	-1.0655024	-0.3010813
INF	-0.2832008	0.08595913	-0.3102122	-0.2184419	-0.2029297	0.26080492
INF	-0.0089472	0.33405725	-0.187115	0	0	0.23478985
INF	-0.0963649	0.10272328	-0.2486478	-0.3131058	-0.2132659	0.41330789
INF	-0.117182	-0.2264512	0.31013856	-0.0252619	-0.4273925	0.22688926
INF	-0.061586	-0.1269264	0.85724676	-0.1034197	-0.3636667	0.10165269
INF	1.88719034	-0.6270376	1.36636168	1.75832651	1.93468362	1.05290615
INF	2.24648719	-0.0954403	1.72565853	2.11762336	2.29398047	1.412203
INF	-0.1079561	0.35475392	0.22656996	0.14699049	0.04982617	-0.4266994
INF	0.04731826	0.35852603	0.17607988	0.15140955	0.08532615	0.12659803
INF	0.13529023	0.02967412	-0.0296508	-0.8790503	0.02353151	-0.3667732
INF	-0.1539412	-0.0018891	-0.1416245	1.80624055	1.98259766	-0.3056672
INF	0.16674047	-0.064258	-0.0897478	1.8185903	-0.0876187	-0.3420004
INF	0.00207287	0.21517612	-0.0913926	-0.1138694	-0.3192299	-0.4287529
INF	0.06826095	0	-0.1846296	1.83046918	2.00682629	-0.3301215
INF	-1.119354	-1.2623587	-1.4219034	2.24981735	-2.0895593	-0.8919792
INF	-1.0026852	-1.0086075	-0.7336891	-1.2442552	2.98564388	-0.5504452
INF	-1.0103749	-0.9432786	-0.7237074	2.19762026	-2.3731505	-0.7062167
INF	-0.9840591	-0.943575	-0.7212717	2.60143492	-1.8443971	-0.2934896

INF	-1.0718198	-1.2089665	-1.0146388	2.56017188	2.89174021	-0.6120159
INF	-1.227051	-1.1998988	-0.984013	2.38063179	-2.2890643	-0.791556
INF	-1.0138468	-1.3428727	-1.020303	2.34434181	-1.4273438	-0.7421291
INF	-0.9494526	-0.3720443	-0.1841542	-0.6789917	-1.3640754	0.30818398
INF	-0.4926499	-0.1826187	-0.1034374	3.05235995	-1.9955321	0.13031052
INF	-1.3165967	-1.4477208	-1.3395663	2.4680539	2.79962223	-0.8487868
UNK	0.04559699	0.20769017	0.1166433	-0.0085214	0.0085214	-0.0798277
UNK	-0.5337117	-0.5320656	-0.702985	2.87182577	3.20339409	-0.1535004
UNK	-0.8324339	-0.8738266	-0.6821893	2.55427662	-1.7154641	-0.4375579
UNK	-0.7859542	-0.4327264	-0.5367312	2.422041	-1.4866718	-0.4728835
UNK	-0.1115758	0.21957945	-0.3672536	-0.0898467	-0.0494009	0.34508478
UNK	-0.0488134	0.21478041	-0.3235144	-0.0602067	-0.0434184	0.28560445
UNK	-0.2635837	0.00010265	-0.3293394	-0.416928	-0.2473189	0.31833567
UNK	0.01012306	0.13454924	0	-0.247102	0.27711927	-0.1934162
UNK	-0.0845516	0.07602108	-0.0704698	1.94462898	0	-0.3297867
UNK	-0.6259374	-0.528612	-0.4385651	2.75502087	-1.5228378	-0.2587777
UNK	-0.5570681	0.39568866	0.17602599	0.19736819	-0.4562408	0.8816081
UNK	-0.8222888	-0.5221799	-0.3519681	2.63084242	-1.3378683	-0.021141
UNK	-1.0679737	-0.9125601	-0.7572408	2.528473	-1.7412678	-0.4424551
UNK	-0.8021777	-0.7054981	-0.6853786	2.44752423	-2.0427269	-0.4301061
UNK	-0.8333979	-0.7302104	-0.5706105	2.75209608	-1.6065858	-0.179262
UNK	-1.1121704	-0.7430871	-0.6528587	-1.1976006	-2.0101208	-0.6501737
UNK	-1.0355525	-0.4584701	-0.3903818	-0.8993792	-1.661631	-0.1330672
UNK	-1.1682347	-1.4763265	-1.4657763	2.43944818	2.7710165	-0.6739454
UNK	-0.9128025	-0.8172957	-0.8055104	2.69488037	-1.7757752	-0.3304458
UNK	-0.8221595	-0.4189982	-0.8287756	-2.5583216	-2.4270426	-0.4774405

CCL5	ICAM	IL1α	IL1β	IFNγ	MPO	CXCL10
0.67968017	2.08915377	-0.3874813	-0.5067947	-0.6000222	0.96797844	0.50985224
0.14704612	-0.3600549	-0.5980936	-0.8035042	-0.7295241	-0.3556082	0.47871859
-0.8191218	1.13439506	0.0584823	-0.4524327	-0.8567058	0.20960958	0.12602523
-0.9861335	1.71509324	-0.0714705	-0.5807513	-0.9576024	0.56022633	0.24178492
-0.4828407	0.90976409	0.04289749	-0.4644398	-0.8199539	0.92715782	0.03003078
0.873568	2.19188459	-0.4728498	-0.4679768	-1.8590364	-0.1015428	1.64805662
	1.69240362	-0.7157497	-0.581503	-0.7852852	1.48304089	0.06096077
		-0.4701658	-0.2781445	-0.8547844	1.61654763	0.12998872
2.25425585		-1.0483415	-1.8127045	-0.3863966	0.70699021	1.83144624
2.01121331	1.69411805	-1.0174939	-1.4098837	-0.6861285	-0.0044263	0.722259
2.50786185	4.21492529	-1.077237	-1.4302366	-0.5742352	0.6661136	1.25255072
2.26171783	1.87329699	-1.434194	-2.0998158	-1.0998158	0.8983677	0.10045166
1.93618352	1.68237445	-0.6234908	-1.1893646	-0.6676806	0.60694283	0.90351793
2.30646822		-1.3361303	-1.6501808	-0.8077886	1.34336279	0.32137741
1.90716169	1.77278776	-1.958257	-1.7936455	-1.4662866	0.88313515	0.07024356
0.44249675	0.11956042	-0.6555872	-0.9905637	-0.8251963	-0.318541	0.54060353
0.52352101	1.8418376	-0.9131149	-0.9621869	-2.2090834	1.98621819	1.29800962
-0.9583689	0.28383213	-0.1934023	-0.1862137	-0.8221269	0.45485026	0.18381501
	1.37849665	-0.4164371	-0.5801393	-0.7434851	1.44296682	0.53002792
		-0.5220299	-0.4472421	-0.7670212	1.17858817	0.4786454
2.71017794	1.9447586	-0.8640345	-1.0143081	-0.5196423	0.89635025	0.97871508
2.13120273		-1.4903559	-2.1824594	-0.9756864	0.54215374	0.78950732
2.10075334	1.99036352	-1.3894643	-2.0702692	-1.0862574	0.7967223	1.24603716
2.31961831		-1.4774345	-1.7618652	-0.8985424	0.96121401	-0.0754524
2.55352237	2.15859723	-1.1846755	-1.6060416	-0.8411725	1.58726412	0.87113201
2.0647649	1.39678801	-1.9085634	-2.4472807	-1.461149	0.44962328	0.25679625
2.70894549	2.65925464	-0.9703982	-1.4263302	0.06848677	1.22670768	1.66672446
1.17611594	2.04910369	0.01034501	-0.6163819	-1.0022702	1.7389892	1.72328463
-0.7403627	0.78286092	0.10270118	-0.3640587	-0.6642363	0.30470425	0.13252845
-0.4393829	2.06738558	-0.0300366	-0.5996799	-0.8788646	1.02984296	1.68629243
-0.8489822	2.09718654	-0.2289297	-0.8741938	-1.2647098	1.35825312	1.46451448
-0.2647414	2.11843371	-0.2473345	-0.7479368	-0.5948738	1.62656194	1.62535082
0.48104308	1.79935967	-1.0458035	-1.0046648	0.98119355	1.94374026	1.25553169
0.84033993	2.15865652	-0.596296	-0.5610744	-1.8922645	-0.7223059	1.61482854
		-0.3317822	-0.5763346	-0.2923585	1.57551983	0.8226479
		-0.3809023	-0.3354551	-0.4196947	0.98454013	0.40305002
	2.02321569	-0.9036607	-0.7934509	-0.9330921	1.40652059	0.09463248
		-0.5812975	-0.5364061	-0.828456	1.28463822	0.30232579
	1.76234631	-0.5524438	-0.5171794	-0.7818499	1.45584084	0.02504124
	1.8905429	-0.3782447	-0.635905	-0.6420948	1.34578463	0.48808038
	1.59575814	-0.4501763	-0.5053005	-0.7936593	1.58504443	0.52966539
2.12666596	1.89776295	-1.6695318	-1.4545567	-1.1618097	0.79284764	0.32141951
2.42658098	1.55118137	-1.3590543	-1.9863311	-1.0394945	0.78166315	0.72750625
2.10414231		-1.6583862	-1.6709753	-1.1165396	0.89849754	0.90360058
2.45738834	1.83265955	-1.4537563	-2.422976	-0.9398017	0.99831613	0.53561304

2.34675039	2.1316318	-1.153043	-2.1302088	-0.8604681	1.13072313	1.01008461
2.24887016	2.81818752	-1.7378413	-2.0900238	-1.330356	0.95551201	0.25436847
2.21626882	2.77098859	-1.7741313	-1.7629231	-1.0021741	1.22443005	0.2757705
2.36976893	2.44311984	-1.1474205	-1.7412602	-0.7980131	1.11747747	1.36879053
2.86246244	2.53479574	-0.898622	-1.3874817	-0.1673019	1.11868961	1.54877031
2.17328642	2.06543621	-1.6889826	-2.2223268	-1.1657937	-0.0027933	0.00279334
0.1803302	1.66559318	-0.4544148	-0.57906	-0.683837	1.41295858	-0.3290091
2.05285714	1.62767531	-1.2552475	-1.3521575	-1.0529408	1.03771706	-0.2663914
2.3594887	2.33819611	-1.6027599	-1.77576	-0.9516203	1.35209231	0.22553168
1.93933149	2.24656622	-1.7138062	-1.7540187	-0.8326817	1.0128157	0.48333367
-0.8013265	1.15568352	0.11205186	-0.4771286	-0.8093321	0.29199926	0.18701662
-0.5333653	0.95246434	0.04341836	-0.4457851	-0.773749	0.3299413	0.40138071
-0.4723235	1.80212262	-0.0001026	-0.6414659	-1.1580957	2.15788323	1.71976909
	2.00505529	-0.1803782	-0.5511402	-0.702928	1.61041265	0.66481865
	2.03933679	-0.4110265	-0.4201312	-0.6847287	1.62856697	0.43026452
2.62764843		-1.0624222	-1.2856925	-0.5087295	1.61200964	0.30256385
1.32771139	0.77755042	-0.9451232	-0.8454707	-0.7952681	-0.0399095	1.04708433
2.27776418	2.1964663	-1.369126	-1.4904785	-0.9415398	1.24237005	0.50292872
2.23470754	2.25325836	-1.6475867	-1.8556134	-1.0520576	0.96063587	0.33687343
2.16941436		-1.4920283	-1.6175942	-0.8504598	0.7855065	1.0877454
2.49311253	2.33835485	-1.3335845	-1.6589738	-0.9588917	1.12415982	0.42770535
2.17913104	2.00559395	-1.5245663	-1.7576961	-1.1570671	0.88751281	0.46358084
2.0610803	1.80231053	-1.2951165	-1.5896047	-1.0055892	1.01523106	1.15461807
1.71912851	1.94629965	-1.6084438	-2.1561462	-1.1410824	0.87253671	-0.1063203
2.3314093	2.56703832	-1.4409668	-1.9955003	-0.8990142	0.78597487	-0.0305626
2.20402552	2.655119	-1.2877112	-1.854272	-0.6685257	1.15895724	1.12548274

Leptin	CCL2	CCL3	IL10	IL6	IL1 RA	IL8
1.82640841	0.15579644	-0.2685488	-0.9478975	-0.3886534	0.46813258	-0.1205527
0.39698152	0.03052272	-1.4014476	-0.4734771	-0.0947192	-0.2744651	-1.6205735
0.65347554	0.48426456	-0.3222065	-0.508082	-0.2301973	0.05818391	-0.4180428
0.40248512	0.24964425	-0.385416	-0.5990514	-0.0708184	0.15769057	-0.2776182
0.54429052	0.30444702	-0.2889811	-0.4938615	-0.2382509	0.01236528	-0.2960015
2.40530203	-0.2000644	-0.4581093	-0.8375867	1.98555861	-0.1978152	-0.0960047
1.08709401	0.23992818	0	-0.85275	-0.1348334	0.66446277	0.58782531
0.77436828	-0.1311928	-0.14995	-0.651763	-0.0716949	0.4028455	0.4432028
0.91666279	0.33218577	-0.7304242	-1.5415166	-0.8753747	0.88181843	-1.6388811
1.73628614	0.00442626	-0.6657771	-1.2337925	-0.8638506	1.03906783	-1.570735
1.57842128	0.46556972	-0.6465995	-1.4452138	-0.4990892	0.41312287	-1.5990286
1.88001651	-0.0247451	-0.6370924	-1.6195588	2.68777418	0.28165245	-1.5835967
1.9424581	0.05236246	-0.8662372	0.04644123	-0.0411621	0.23958858	-1.7043612
0.90529221	0.02758482	-0.1755845	-1.3548528	-1.6174569	0.45658641	-0.683529
-0.0198243	-0.5929318	-1.0453652	-2.1381412	2.43139301	-0.1748506	-2.5004409
0.53600667	-0.0171355	-1.3023176	-0.5064376	-0.1152671	-0.2776951	-1.6180708
2.05525504	-0.6311028	-0.8494295	-1.4055265	1.63551162	-0.5990967	-0.4732191
0.89290101	0.04069952	-0.3664681	-0.4425827	0.1403292	0.04361314	-0.0406995
1.72947574	0.15420753	-0.3027101	-0.8151806	-0.1202074	0.5211535	-0.0009616
1.30763865	0.34554918	-0.190977	-0.7348509	1.69181453	0.22372983	-0.0116942
1.17838346	-0.0095767	-0.9061879	-1.7671245	-0.246772	-0.0866077	-1.1585985
1.25049731	0	-0.9545223	-2.1910596	2.56031823	-0.2505431	-1.317158
1.71945075	-0.6986451	-0.8155785	-2.4979856	2.46100047	-0.0283536	-1.9599636
0.88473453	0.15026823	-0.8462895	-1.4413727	-0.7654995	0	-1.5833679
1.52646887	-0.5195102	-0.4666936	-1.4916959	2.92522816	0.20022016	-1.0642218
1.03687266	2.3192815	-1.218294	-2.4115651	2.45899908	-0.3774013	-2.4350462
0.99533643	0.03961892	-0.1834103	-1.2656759	-0.3658122	0.78843802	-0.9533649
0.33691959	0.26018085	-0.3911621	-0.6034169	-0.3420041	-0.010345	-0.1851574
0.7125553	0.41166833	-0.2479548	-0.4393327	-0.1775697	0.05209446	-0.1160517
0.38255543	0.08563523	-0.4545378	-0.4299542	0.02749081	0.68268682	-0.0274908
0.04034993	0.664247	-0.6493602	-0.8537182	-0.3080206	0.5217047	0.02526187
-0.0066483	0.26541476	-0.5260881	0.00664835	0.22731153	1.60851994	0.67093995
2.01277711	-0.7545899	-0.9266157	-1.4480045	1.59303369	-0.7076496	-0.5531277
2.37207396	-0.3142839	-0.4853598	-0.9571559	1.95233054	-0.2683515	-0.1292327
0.89383701	0.49357462	-0.3062525	-0.3545116	0.01488163	0.68759858	-0.1944104
1.01101448	0.02672513	-0.2673953	-0.6929801	0.07840812	0.1588186	-0.2463774
1.08295326	-0.1050357	-0.4484108	-0.7006376	0.12679992	0.14915912	0.18632247
1.52870151	0.35470607	-0.3605464	-0.8186988	-0.7084211	0.17589569	-0.0611719
1.21894707	0.28716356	-0.2958082	-0.4137392	-0.0730628	0.78368953	0
1.78094679	0.22327958	-0.2981465	-0.7519429	0.07013733	0.51628743	0
2.08491978	0.20065583	-0.2612906	-0.7614891	1.66517636	0.71901292	-0.1275132
1.47600105	0.14585932	-0.7613127	-1.5092791	-1.5909492	0.7186443	-0.4577407
1.34991537	0.04735481	-0.7625982	-1.4672454	2.80125886	-0.1854946	-0.3774797
0.10287024	-0.0522353	-0.8763745	-1.1740864	-0.5216656	0.52778279	-0.8454729
1.60663844	0.04162336	-0.8516963	-1.5587148	2.74861823	-0.1206906	-1.5142089

0.89842198	-0.8667748	-0.8966555	-0.9085719	-0.9721406	0.13270513	-0.7421249
0.77020847	-0.1840693	-0.8663905	-1.6920838	2.52781509	0.01803357	-0.6862474
0.39332121	-0.1125475	-0.6685654	-1.6823609	-1.4964247	0.75802336	-0.0799564
-0.1887948	-0.2443305	-0.6878616	-1.2055442	-1.2119154	0.32816123	-0.101193
2.16950443	0.09003599	-0.2680812	-0.8292006	-0.9306583	0.78157242	-0.7899067
1.26160132	-0.1110806	-1.1141249	-3.1361406	2.61523721	0.02922245	-2.0289306
2.10299744	0.21510265	-0.1237891	-0.5588436	0.21153393	0.66642856	0.15926566
1.90186461	-0.1878046	-1.0989003	-1.2494952	-1.3379171	0.79285753	-1.4313387
1.65420516	0.51270801	-0.916379	-0.3924796	-0.6671806	0.41495899	-1.3457674
1.20759361	0.32180052	-0.6576276	-1.1147107	-0.6224862	0.83553078	-0.3339644
0.62292849	0.56065996	-0.3073499	-0.5103268	-0.2513496	0.04940088	-0.3672536
0.64883621	0.55353732	-0.3014571	-0.5058547	-0.2400961	0.45023513	-0.5193922
0.93147303	1.0409539	-0.3518234	-0.6939692	1.24579486	1.00016421	0.48042753
1.45669896	0.2714762	-0.1528543	-0.7677988	-0.4703008	0.88696662	-0.1473438
1.76764331	-0.0719781	-0.0842829	-0.7233835	-0.0633214	0.52992074	0.26165714
1.22789616	0	-0.302631	-1.0040756	-0.8338139	0.64949524	0.25360298
0.10176081	0.08091125	-0.3302514	-0.1098297	0.03990952	0.51892058	-1.2410737
0.36218604	-0.3338656	-0.3663262	-1.0982908	2.77802572	0.1974375	-1.3398836
0.80831445	-0.0374193	-0.5914217	-1.6955103	2.67565631	-0.2176658	-1.1422342
2.59905628	-0.0510239	-0.6589116	-1.3267236	-1.2897925	0.99315212	-1.5375769
0.11759698	-0.2382566	-0.7256423	-2.4718872	2.89927938	-0.0972916	-1.1133179
0.89610017	-0.1920502	-0.4460816	-1.5721838	-1.7168232	0.30113435	-0.8482245
0.28819888	0.20656377	-1.0299486	-1.4941399	-0.9214232	0.33348776	-1.3145882
0.32756697	-0.8742665	-1.1427306	-1.6488725	2.58663148	0.03926506	-1.3208909
2.08594802	-0.3479742	-0.8103245	-1.3318223	2.84206368	-0.0049232	-1.9093141
2.45060558	-0.0135242	-0.9062459	-1.3671666	-0.9562616	0.28222731	-0.716153

VCAM	TNFα	IL4	TRAIL	MMP8	MMP9
1.09802879	-0.6573891	-0.2205031	0.35378614	-2.3556387	-2.4246351
-0.171481	-0.2732435	-0.2145659	0.12882461	0.42192819	0.16252575
1.90504794	-0.3674864	-0.1961941	0.10429944	0.29878462	1.40224516
1.80134446	-0.4648662	-0.2834265	0.07081837	0.32075463	1.03144082
1.11336799	-0.3478784	-0.1715456	0.2977767	0.38025168	0.88715232
-0.6341571	-0.6239152	-0.1914893	1.92956773	-0.9417545	0.54814467
0.91660175	-0.5546423	-0.35937	0.14937879	-0.2880996	0.69683388
0.86852445	-0.3730725	-0.1040771	0.23625985	-0.4903763	0.63859371
1.95412354	-1.2966652	-1.132454	0	-0.1736696	0.38715347
1.75334391	-0.9852095	-0.6966733	0.25845779	0.05841669	0.82131471
2.1966559	-1.1403908	-0.760967	0.95269575	-0.1865681	1.54122007
1.9256744	-1.0601431	-1.067092	0.02474513	0.26121365	1.38512742
1.85164786	-1.4884898	-0.4933636	-0.1070528	-0.2542561	0.04021318
2.2120476	-0.4786451	-0.819899	0.28466883	-0.0578592	1.69373105
1.70314744	-1.6687068	-1.4201643	0.01982426	0.51625067	1.58623707
-0.0813383	-0.324594	-0.2466845	0.01713548	1.24445121	1.09309617
-1.0832294	-1.5318719	-0.6659697	1.57952074	-0.8387377	0.13544499
-0.3170342	-0.2128513	0.07895453	0.12214433	-2.2028317	-2.5685568
1.0279581	-0.6766285	-0.3823859	0.30471164	-0.5696182	0.65485374
1.1751855	-0.5517199	-0.2222369	0.37528581	-1.3746292	-0.6596228
2.5410637	-0.9918783	-0.5470164	0.14732442	-0.1782928	1.06080019
1.53273584	-1.6832037	-1.2555523	-0.1285528	0.07437148	1.1685217
1.51683151	-1.7198344	-1.372347	0.02835363	-0.0760104	1.2206687
1.94958628	-1.4032959	-1.1126927	0.15168374	0.11454508	0.90475415
2.06692046	-1.1503713	-0.9081193	0.41619297	-0.6787825	2.22355358
1.24944255	-1.8390604	-1.4104238	0.28216181	0.01984095	1.84859981
2.35356304	-1.2069702	-0.822207	0.7257251	-0.1983211	-1.0953369
2.12866587	-0.5438312	-0.3028433	0.53792747	0.75436587	4.74657081
1.50199062	-0.2578631	-0.0953447	0.24540364	-0.7966186	0.24853784
2.19204277	-0.4986533	-0.29054	0.16490538	0.3405519	3.93597927
2.09202021	-0.7754904	-0.5827535	0.10828535	0.62637699	4.26784481
1.67446571	-0.6862649	-0.4931177	0.49294039	-0.0985928	1.01229799
-1.1257074	-1.5743498	-0.7339101	1.53704281	-1.1460726	-0.0591245
-0.7664105	-0.9133909	-0.2535992	1.89633966	0.3347929	-0.9443273
1.02009855	-0.3718696	-0.2512684	0.63850295	-0.6421258	-0.2879358
0.88884057	-0.3204825	-0.2260939	0.49417524	-1.6694791	-0.7715856
0.87319347	-0.5559217	-0.5253422	0.1141936	-0.5459087	0.27359554
1.05084783	-0.6801236	-0.2432376	0.40495285	-1.5509212	-1.1072627
1.04891712	-0.6815061	-0.2379798	0.42291677	-0.7875764	0.83443487
0.79493155	-0.6347444	-0.3097898	0.25489638	-0.4264548	-0.0710618
0.98656046	-0.7000062	-0.2655181	0.27379183	-1.4440571	-0.0007207
1.71038997	-1.7270113	-1.3730116	-0.1217876	0.1217876	0.96204613
1.83003124	-1.4085397	-1.0502984	0.33786183	-0.0473548	0.83031358
1.60055792	-1.5078491	-1.3581324	0	0.12216254	1.25624023
2.00844365	-1.263979	-1.1208046	0.06214744	0.46083482	2.09533704

1.99993429	-1.5024434	-1.1259923	-0.0063571	1.05806103	1.60072311
1.86981344	-1.6530198	-1.3237422	-0.0180336	1.36343619	1.62871106
1.65310019	-1.6893098	-1.2858018	0.07995637	0.14123752	1.68758691
2.00028993	-0.8839277	-1.1249598	0.10119303	0.5481987	1.38327393
2.4190588	-0.7765883	-0.6254921	0.55868782	-1.0333206	3.02384045
1.77050146	-1.7047768	-1.340956	0.57331593	2.4807586	-1.6072239
0.66677647	-0.3745163	-0.2116561	0.34680526	-2.3075784	-2.7987104
1.44449777	-0.9749727	-0.7220688	0.0918873	-0.0918873	2.36916939
2.2932669	-1.3741395	-1.0534062	0.24810509	-0.2340746	1.41602383
1.6950911	-1.0113053	-1.200788	0.06177703	-0.1411558	0.50197342
1.7444324	-0.3564407	-0.211841	0.21768544	0.45007283	1.71197021
1.6047781	-0.3148427	-0.1610153	0.18481719	0.28434589	2.55727417
2.02723636	-0.6136058	-0.4094858	1.21043786	-0.0528752	0.35673206
1.01825042	-0.5302104	-0.2799764	0.41330003	-1.0619833	0.54703849
1.23487538	-0.6027854	-0.2106928	0.4063672	-1.3214293	0.10316219
1.99912882	-1.1733953	-0.3383606	0.66516157	-0.0879978	0.96585925
0.94126032	-0.1384931	-0.4896697	0.05560983	-0.5997924	-0.1481284
2.25800024	-1.0249391	-0.9014701	0.41744176	0.02114103	2.13747995
1.93249956	-1.3004752	-1.1166801	0.0559613	-0.1046313	1.2634118
1.93356802	-1.2850973	-1.1272865	0.13563011	-0.0756832	1.74921623
2.26375096	-1.1344279	-0.9425424	0.3227751	0.49845969	1.86098367
1.75753877	-1.3418445	-1.2739587	-0.0564463	0.54513022	1.17559135
1.77748609	-0.8610757	-1.1418288	-0.0769008	0.23565099	1.14074825
1.66389423	-1.6934546	-1.302615	-0.083327	0.31428163	1.23322842
2.04964612	-1.3094311	-0.9579766	0.23050201	1.54341047	1.94274966
1.96685972	-1.5192575	-1.0932888	0.30444101	0.01352423	1.27314545
