# Supplementary material for: Characteristic Cytokine and Chemokine Profiles in Encephalitis of Infectious, Immune-Mediated, and Unknown Aetiology
Source: PLoS One. 2016 Jan 25;11(1):e0146288. doi: 10.1371/journal.pone.0146288 (PMC4726626; doi:10.1371/journal.pone.0146288)
Supplement: S2 Table — (PDF) [file pone.0146288.s002.pdf]

		G CSF	GM CSF	Granzyme B	IFNa2	IFNb
CSF	ADEM	2.36027561	0.116191496	1.839446952	2.231411776	0.322376084
CSF	IMM	2.304106876	0.016212784	1.783278219	2.175243042	0.257367494
CSF	IMM	-0.268839405	-0.210236949	-0.358012391	1.74034886	1.916705977
CSF	ANT	-0.138537346	0.181552775	-0.374704793	-0.058183914	-0.058183914
CSF	ADEM	0.771478059	0.076657322	-0.381400927	-0.141035468	-0.167484103
CSF	ANT	0.148313604	0.34549398	-0.049541038	0	0.202198546
CSF	ANT	-0.054897935	0.258330612	-0.350788291	-0.012365281	-0.06649977
CSF	INF	2.218440355	0.011200365	-0.495335088	2.089576521	2.265933638
CSF	HSV1	0.274555964	-0.059139138	0.323379651	0.463375068	1.881776533
CSF	HSV	1.96622378	-0.24101621	1.445395123	1.837359946	2.013717063
CSF	HSV1	2.141137649	-0.006343285	1.620308992	-1.12827882	2.188630932
CSF	HSV1	0.167336659	-0.169612621	0.216454222	0.116638188	1.86854353
CSF	HSV1	0.058493772	0.012689861	0.069937173	-0.637049181	2.037800739
CSF	INF	0.348565673	-0.074407784	-0.218592712	-0.831037151	1.957136528
CSF	INF	1.887190341	-0.627037634	1.366361684	1.758326507	1.934683624
CSF	VZV	-0.272520946	0.078814168	-0.375183288	-0.244744401	-0.212349559
CSF	HSV	-0.096364893	0.102723283	-0.248647829	-0.313105818	-0.213265871
CSF	VZV	-0.117181961	-0.226451231	0.31013856	-0.025261874	-0.427392507
CSF	HSV	-0.06158595	-0.126926439	0.857246761	-0.103419661	-0.363666744
CSF	HSV	2.069151467	-0.030835929	-0.092135754	-0.127629646	2.11664475
CSF	UNK	1.94327802	-0.263961971	1.422449362	1.814414186	1.990771302
CSF	UNK	0.160879151	-0.147636672	-0.232751379	-0.419016616	1.942977862
CSF	UNK	-0.177243558	-0.201856146	-0.329093835	1.769267416	1.945624533
CSF	UNK	-0.345877379	-0.133961038	1.520655974	1.912620798	2.088977914
CSF	UNK	-0.231393329	-8.11753E-05	-0.213360561	-0.783078124	2.090533358
CSF	UNK	2.090758504	-0.423469471	1.569929847	1.96189467	2.138251787
CSF	UNK	-0.422802867	-0.159116511	-0.48855852	-0.576147156	-0.406538024
CSF	UNK	-0.005610144	0.145963873	-0.41836324	-0.270314317	-0.198233012

IFNo	CCL5	sICAM	IL1a	IL1b	IFNy	MPO
-0.367943162	0.954128345	2.272444934	-0.31045628	-0.321047974	-0.894821763	0.805324975
-0.586510853	0.897959612	2.216276201	-0.384449986	-0.3970859	-1.041794585	0.52083376
-0.696559151	-0.625291422	1.46549903	-0.367619702	-0.564000541	-0.589564441	0.704922843
0.289071562	-0.81912177	1.134395059	0.058482296	-0.452432738	-0.856705812	0.209609582
0.170961188	-0.986133508	1.715093243	-0.071470467	-0.580751306	-0.957602419	0.560226333
0.109323819	-0.757654422	0.785885283	-0.145321546	-0.245060849	-0.616838923	0.233477355
0.162048516	-0.482840736	0.909764088	0.04289749	-0.464439779	-0.819953887	0.927157821
-0.509778416	-0.165425959	1.216298658	-0.007592772	-0.372028662	-0.530431395	0.930458349
-0.568996214	-0.583341264	1.224670261	-0.211218787	-0.513490553	0.000771929	1.299208832
-0.761994991	-1.14374772	0.621961925	-0.632069167	-0.672380615	-0.960011545	0.05996255
-0.587081122	-0.260195023	1.017180987	-0.271042812	-0.475468823	-0.662082848	0.910673786
-0.554622487	-0.465050906	1.281300531	-0.386978151	-0.61808646	0.041448026	0.95226681
-0.338316687	-0.209472264	1.449665021	-0.043878835	-0.442905778	-0.625084389	0.98757112
-0.379811252	-0.568615703	1.612609672	-0.122048031	-0.307356436	0.566120879	1.204865657
1.052906149	0.481043077	1.799359666	-1.045803515	-1.004664784	0.981193548	1.943740263
0.136306869	-0.690642999	1.399901012	0.005264433	-0.615392046	-0.998773335	0.540357933
0.413307889	-0.439382905	2.067385583	-0.030036646	-0.599679897	-0.87886456	1.029842962
0.226889259	-0.84898216	2.097186539	-0.228929702	-0.874193828	-1.264709819	1.358253119
0.101652691	-0.264741439	2.118433711	-0.247334491	-0.747936842	-0.594873817	1.626561942
-0.421965983	0.453329967	1.437231197	0.014503139	-0.40235902	-0.498907301	1.390638517
-0.784940752	-1.107092041	0.850178876	-0.541241312	-0.647190997	-0.883773144	0.141882504
-0.575238131	-0.510630289	1.095417176	-0.235434416	-0.470657199	-0.726580211	1.393894218
-0.667640595	-0.650215471	1.226122306	-0.196639825	-0.603402989	-0.715477647	0.674387133
-0.524287214	0.635337367	1.207924826	-0.41843686	-0.514986075	-0.661048283	0.290767925
-0.427682634	-0.515223254	1.460984559	-0.117389601	-0.466718188	-0.545683574	0.534827722
1.256474312	0.68461124	2.002927829	-0.792599117	-0.80109662	1.184761711	2.147308426
0.159116511	-0.631542645	1.642903464	-0.159321803	-0.800685044	-1.31731484	1.998664069
0.392613934	-1.036013365	1.260420879	-0.165890222	-0.600623559	-0.92856536	1.888924543

IP10 (CXCL10)	Leptin	MCP1 (CCL2)	CCL3 (MIP1a)	IL10	IL6	IL1 RA
1.728616962	2.485862376	-0.079418538	-0.136405426	-0.488441437	2.066118956	0.156861128
1.672448229	2.429693643	-0.175672742	-0.198867391	-0.586109458	2.009950223	0.097186969
0.672686573	1.994799461	0.159720967	-0.406868462	-0.855492246	-0.498214404	0.114974246
0.126025235	0.653475543	0.484264564	-0.322206455	-0.508081983	-0.230197261	0.058183914
0.241784921	0.402485123	0.249644247	-0.385416004	-0.599051427	-0.070818373	0.157690573
-0.049541038	0.629053207	0.782701681	-0.174479775	-0.384429301	-0.13308709	-0.009796532
0.030030781	0.544290524	0.304447023	-0.288981141	-0.493861488	-0.238250879	0.012365281
1.104421413	2.344027121	0.081124852	-0.176225331	-0.605923976	1.924283702	0.354035617
1.202624603	1.959870017	0.6828905	-0.130083397	-0.000771929	0.979238871	1.354952484
-0.05996255	2.091810546	0.290906241	-0.536750487	-0.870023567	1.672067127	-0.132799177
0.757096255	2.266724416	0.673692932	-0.279889022	-0.671866798	1.846980996	0.535725443
1.045973076	0.941386422	0.238675533	-0.383463802	-0.236283356	0.441146632	1.134512534
1.35864881	1.100468405	0.608815063	-0.27412015	-0.662926596	0.660578387	0.535701062
1.200638581	2.035230012	0.595655768	-0.101889337	-0.484844996	0.956810714	1.321400731
1.255531694	2.012777107	-0.754589864	-0.8919074	-1.448004451	1.593033688	-0.707649578
1.173531215	0.370360934	0.63023277	-0.453343865	-0.611461753	-0.333532631	0.203253658
1.686292434	0.38255543	0.085635226	-0.454537775	-0.429954161	0.027490815	0.682686823
1.464514477	0.040349931	0.664246999	-0.649360225	-0.853718243	-0.308020574	0.521704698
1.62535082	-0.006648348	0.265414762	-0.526088092	0.006648348	0.227311531	1.608519943
1.324382342	2.194738234	0.458569852	-0.175009334	-0.556003568	-0.067662774	0.210240232
0.313854178	2.068864786	0.465649723	-0.53150465	-0.905439027	1.649121366	-0.068630158
0.92124422	2.021071346	0.67392548	-0.236203283	-0.569230066	1.246464942	1.096125782
1.08199643	0.837775226	0.64193137	-0.420289879	-0.711561324	0.38830358	0.445913433
0.403362384	2.167071398	0.670234649	-0.383763788	-0.730573609	0.466884551	0.10267941
1.050541848	2.168626842	0.511366476	-0.336643441	-0.685625249	8.11753E-05	0.164717293
1.459099857	2.216345271	-0.470012539	-0.659698631	-1.244436288	1.796601851	-0.504081415
1.560549929	0.77225387	0.881734741	-0.511042551	-0.853188405	1.086575707	0.840945058
-0.068432548	0.406677233	1.094715294	-0.405686615	-0.629522986	0.469374648	0.005610144

IL8	sVCAM	TNFa	IL4	TRAIL	MMP8	MMP9
0.106071075	-0.483338429	-0.677905862	-0.04206951	0.04206951	-3.423024046	-2.962293207
0.03319511	-0.560754681	-0.734074595	-0.141633691	-0.155093319	1.581403924	-4.132405293
0.156312154	1.055639494	-0.825439081	-0.393334216	-0.006714421	-2.938910545	1.105285536
-0.41804281	1.905047944	-0.367486436	-0.19619406	0.104299438	0.298784623	1.402245163
-0.277618186	1.801344459	-0.464866247	-0.283426466	0.070818373	0.320754626	1.031440816
	1.195714514	-0.113781935	0.088103139	0.045918908	-0.456624426	0.588532028
-0.296001484	1.113367987	-0.347878434	-0.171545565	0.297776702	0.565245205	2.483934743
0.33128708	1.143290967	-0.543050023	-0.131765384	0.088455073	-2.316341122	1.454513197
0.898954997	0.887261634	-0.685524994	-0.283617841	0.216793221	-0.645448391	-0.023618357
0.147778021	0.554619874	-0.795266598	-0.43612134	-0.338645669	1.243520828	1.202296622
0.269814867	0.88053647	-0.573605111	-0.225087236	-0.002697653	1.418434697	-4.29537452
0.457732027	0.785747281	-0.794177719	-0.441496662	-0.136315071	1.098347295	-3.686042996
1.319454903	1.41894814	-0.588254389	-0.164909938	0.235468343	-1.662479745	-0.757985175
1.002581644	1.074161113	-0.130835303	-0.236372424	0.270634056	-2.973592877	-1.370850092
-0.553127675	-1.170243037	-1.574349834	-0.708447612	1.537042808	-2.835411474	-4.549321828
-0.005264433	1.640598206	-0.528062447	-0.328915163	0.082589802	0.246712216	0.753612855
-0.027490815	2.192042768	-0.498653318	-0.29053999	0.164905385	0.340551898	3.935979272
0.025261874	2.092020213	-0.775490443	-0.582753525	0.10828535	0.626376993	4.267844812
0.670939952	1.674465708	-0.686264949	-0.493117671	0.492940388	-0.131833773	3.088649344
0.317955488	1.120953062	-0.574461465	-0.226719123	0.138612976	-0.583832292	-0.950678597
0.068630158	0.680826385	-0.793569829	-0.4590671	-0.293115841	1.220575067	-2.53899164
1.101664406	0.812262895	-0.719743387	-0.282857413	0.45634241	-1.930367427	-1.439022902
0.746388094	0.831165174	-0.778146972	-0.369625705	0.071845265	1.175428298	1.134204092
0.550081449	0.960828969	-0.720005747	-0.308721107	0.076964202	1.318781679	-3.373838238
0.309219028	1.036794094	-0.572187891	-0.24690998	0.112501584	1.320337123	1.279112918
-0.329075423	-0.922139207	-1.370781671	-0.504879448	1.740610972	-3.275715798	1.326831346
0.321208374	1.868017201	-0.772824945	-0.568704963	1.051218701	4.094373705	2.873013618
0.789416549	1.930402847	-0.458776957	-0.276455957	0.688645692	0.61191839	1.5783287
